# Removal of prolyl oligopeptidase reduces alpha-synuclein toxicity in cells and *in vivo*

**DOI:** 10.1038/s41598-018-19823-y

**Published:** 2018-01-24

**Authors:** Reinis Svarcbahs, Ulrika H. Julku, Susanna Norrbacka, Timo T. Myöhänen

**Affiliations:** Division of Pharmacology and Pharmacotherapy, University of Helsinki, Viikinkaari 5E, P.O. Box 56, 00014 Helsinki, Finland

## Abstract

Prolyl oligopeptidase (PREP) inhibition by small-molecule inhibitors can reduce alpha-synuclein (aSyn) aggregation, a key player in Parkinson’s disease pathology. However, the significance of PREP protein for aSyn aggregation and toxicity is not known. We studied this *in vivo* by using PREP knock-out mice with viral vector injections of aSyn and PREP. Animal behavior was studied by locomotor activity and cylinder tests, microdialysis and HPLC were used to analyze dopamine levels, and different aSyn forms and loss of dopaminergic neurons were studied by immunostainings. Additionally, PREP knock-out cells were used to characterize the impact of PREP and aSyn on autophagy, proteasomal system and aSyn secretion. PREP knock-out animals were nonresponsive to aSyn-induced unilateral toxicity but combination of PREP and aSyn injections increased aSyn toxicity. Phosphorylated p129, proteinase K resistant aSyn levels and tyrosine hydroxylase positive cells were decreased in aSyn and PREP injected knock-out animals. These changes were accompanied by altered dopamine metabolite levels. PREP knock-out cells showed reduced response to aSyn, while cells were restored to wild-type cell levels after PREP overexpression. Taken together, our data suggests that PREP can enhance aSyn toxicity *in vivo*.

## Introduction

Prolyl oligopeptidase (PREP; EC 3.4.21.26) is a highly conserved enzyme^1^ that was first discovered in the human uterus^2^. PREP is a member of the serine protease family^3^ that selectively hydrolyzes oligopeptides that do not exceed 3 kDa^4^ by cleaving peptide bonds at the carboxyl group of internal proline residue^5^. Ubiquitous PREP distribution has been described in the peripheral tissue^6^, however the highest activity of PREP can be seen in the brain^7–9^ where PREP is highly expressed in the nigrostriatal tract and cortical brain areas^10^. Studies in PREP knock-out (PREPko) animals demonstrated that these animals have impaired dopamine transporter (DAT) function and elevated extracellular dopamine (DA) amount, associated with delayed DA reuptake from the synaptic cleft^11^. Moreover, small molecule PREP inhibitor, KYP-2047, was able to increase locomotor activity in young rats after acute administration^12^. Systematic behavioral phenotyping has demonstrated that PREPko animals are hyperactive and have reduced anxiety-like behavior^13^ that in part could be explained by increased extracellular DA amount^11^.

Recently, PREP has been implicated in the α-synuclein (aSyn) aggregation process. The presence of PREP was sufficient to increase the aggregation rate of aSyn in a cell free model^14^. Cellular data showed that catalytical PREP inhibition disrupts aSyn and PREP co-localization that is accompanied by reduced aSyn aggregation in cells^15^. It was followed by evidence of direct PREP and aSyn interaction and increased aSyn dimerization^16^. A similar line of evidence has been seen in transgenic aSyn mouse models where PREP inhibition was able to reduce aSyn overload^15,17^ and in aSyn viral vector overexpression Parkinson’s disease (PD) model that showed a relationship between aSyn oligomer numbers and restoration of motor behavior after PREP inhibition^18^. In transgenic A30P aSyn mouse model, PREP inhibition was able to increase striatal DA amount^17^, which could be explained by improved aSyn and SNARE complex assembly and neuronal trafficking^19,20^, notably during the aSyn nucleation phase^21^.

In this study, we wanted to clarify the significance and impact of PREP for aSyn aggregation *in vivo*. After viral vector delivery of aSyn or combination of aSyn and PREP (aSyn + PREP) above *substantia nigra* (SN) of wt and PREPko mice, we have measured behavioral changes in mice followed by a set of immunohistochemistry (IHC), no-net-flux microdialysis and high-performance liquid chromatography (HPLC) tissue analysis and supportive cellular data using PREPko cells. Our results revealed that even unilateral delivery of PREP above SN could restore animal motor behavior, however PREPko animals seem nonresponsive to aSyn-induced unilateral toxicity when aSyn viral vector is delivered without the PREP viral vector.

## Results

### Locomotor activity in PREP ko animals is restored to the wt animal levels after PREP and aSyn viral vector co-injection

**Figure 1:**
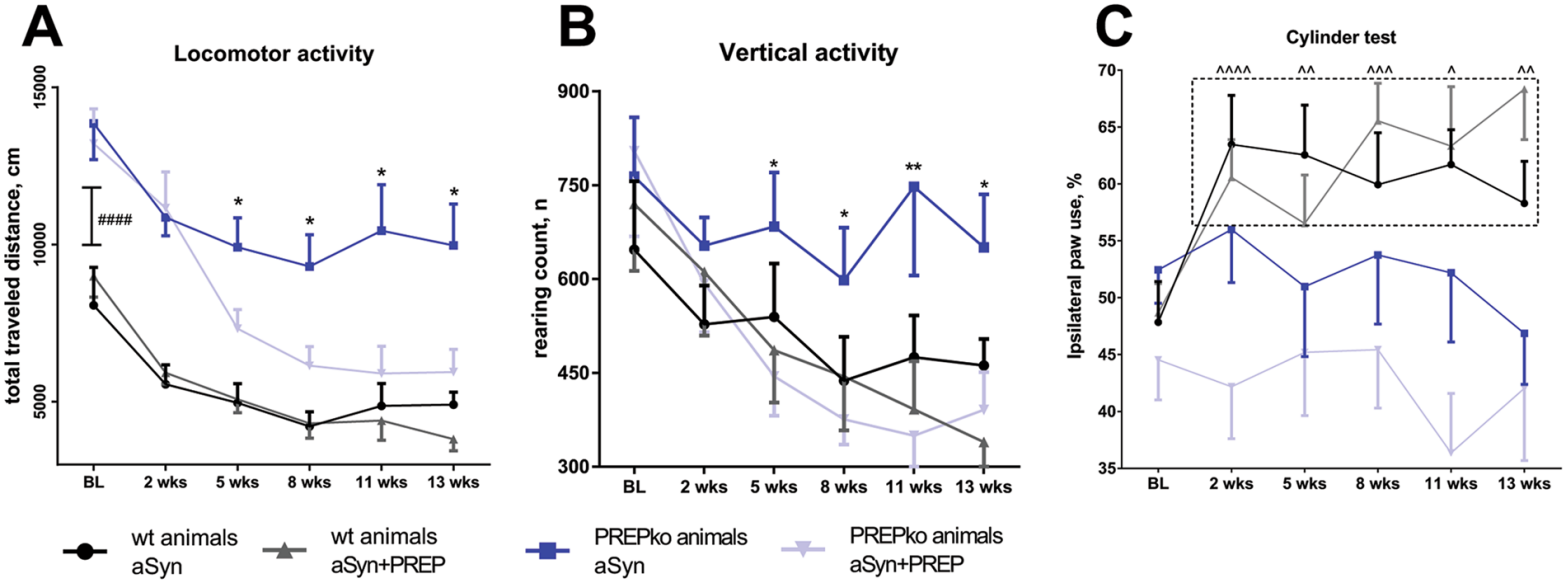
PREPko mice after viral injection of aSyn showed behavioral resistance to aSyn toxicity. (**A**) Total traveled distance was significantly reduced in PREPko animals with aSyn + PREP injection compared to PREPko animals with only aSyn at the 5-week time point and the difference extended until the end of the experiments. BL locomotor activity was drastically higher in PREPko compared to wt animal groups (n = 7–10). (**B**) Similar to total distance travelled, vertical activity was statistically different between PREPko animal groups starting from the 5-week time point and the difference extended until the end of the experiments (n = 7–10). (**C**) Unilateral aSyn viral vector injection caused increased ipsilateral paw use 2 weeks after injection only in the wt animal groups (n = 15–17), and this difference was not seen in PREPko animals. Bars represent mean ± SEM. *p < 0.05, **p < 0.01, PREPko aSyn vs. PREPko aSyn + PREP; ^####^p < 0.0005, wt vs. PREPko; ^p < 0.05, ^^p < 0.01, ^^^p < 0.001, ^^^^p < 0.0005, wt animal BL vs. post-injection measurements (2-way ANOVA with Univariate analyses; Student’s t-test for BL locomotor activity).

There was a statistically significant interaction between the aSyn and aSyn + PREP viral vector injections and time on total traveled distance in the PREPko animal groups (Fig. 1A; F_(5,75)_ = 4.174, p = 0.002, 2-way ANOVA). Traveled distance was decreased in the PREPko animal group that received aSyn + PREP viral injection at 5-week time point (F_(1,15)_ = 5.612, p = 0.032, Univariate analyses) and viral vector effect extended until the end of the experiment at 13-week time point (F_(1,15)_ = 7.642, p = 0.014). A similar effect was not observed in wt littermates (Fig. 1A; F_(5,70)_ = 1.002, p = 0.395, 2-way ANOVA). All animal groups exhibited decreased locomotor activity when compared to baseline (BL) levels from 5-week time point onwards (locomotor activity vs. BL; wt p = 0.001; PREPko animals p < 0.0005). In this experimental setting, we wanted to assess the effect of PREP on aSyn overexpression and therefore the green fluorescent protein (GFP) injected animal groups were deemed redundant. Additionally, it has been previously reported that aSyn can decrease locomotor activity comparatively to GFP viral vector injections^22^.

PREPko animals exhibited higher BL locomotor activity compared to the wt littermates (Fig. 1A; t(_31_) = 1.091, p = 0.000031, Student’s t-test) and this observation was in accordance with the previous^13^ and our group’s observation that PREPko animal show increased activity in the exploratory phase^11^.

### Vertical activity

Similar to travelled distance, there was a statistically significant interaction between the viral vectors and time on vertical activity for PREPko animal groups (Fig. 1B; F_(5,75)_ = 2.539, p = 0.036, 2-way ANOVA). A similar effect was not observed in the wt littermates (Fig. 1B; F_(5,70)_ = 1.161, p = 0.337). Follow up univariate analyses revealed that mean vertical activity was decreased in the PREPko animal group that received aSyn + PREP viral injection compared to the PREPko animal group with aSyn injection. Statistical differences between PREPko groups were seen at the 5-week time point (Fig. 1B; F_(1,14)_ = 6.832, p = 0.02) and treatment effect extended until the end of the experiment at the 13-week time point (Fig. 1B; F_(1,14)_ = 5.052, p = 0.041).

### Cylinder test

There was no statistically significant interaction between the viral vector injections and paw preference either in PREPko animal groups (Fig. 1C; F_(5,145)_ = 0.639, p = 0.622, 2-way ANOVA) or wt littermates (F_(5,150)_ = 1.696, p = 0.139). Nevertheless, the main effect over time showed a significant change of paw preference in wt animals (Fig. 1C; F_(5,150)_ = 5.453, p = 0.001, 2-way ANOVA with Bonferroni’s adjustment). Wt animals exhibited paw preference misbalance that is indicative of aSyn caused toxicity^18^. Paw preference was significantly different in wt animal groups 2 weeks post-injections (p < 0.0005) and remained statistically different to the BL scores for the duration of the experiments (Fig. 1C; p = 0.007, 13-week time point).

### aSyn oligomer-specific staining distribution in SN

**Figure 2:**
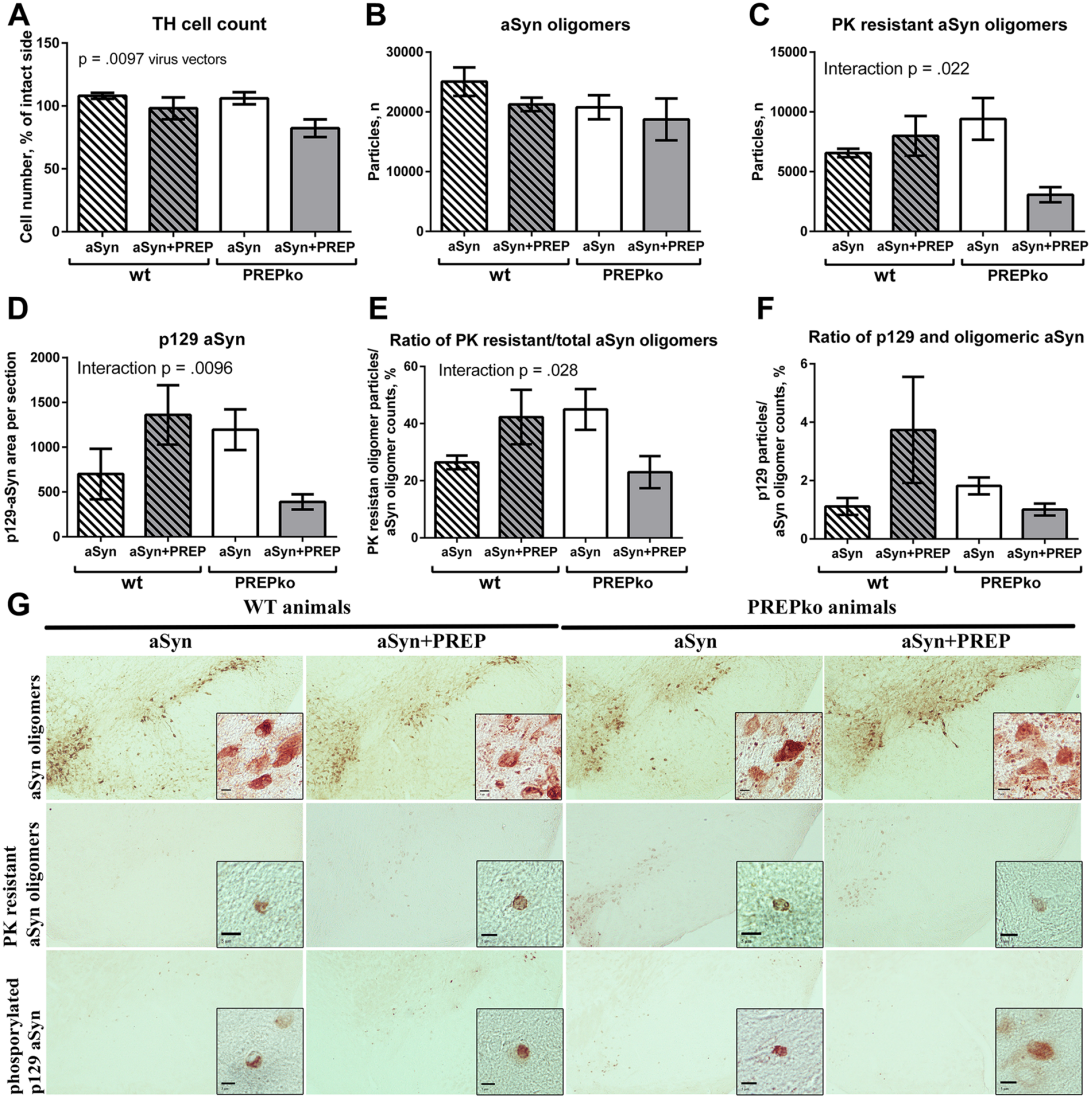
TH+ cells, aSyn oligomer particles and of p129-aSyn OD measurements showed alteration in PREPko animals. (**A**) Significant TH + cell decrease was observed between aSyn injected wt and PREPko animal groups and aSyn+ PREP injected PREPko animal group (n = 6–7). (**B**) Oligomer specific particle stereological counts were not different between the animal groups (n = 7–8) while (**C**), PK resistant aSyn oligomer stereology showed a statistically decreased number of PK resistant oligomers in the PREPko aSyn + PREP animal group (n = 4–6). (**D**) p129-aSyn phosphostaining showed a significant interaction and decreased immunostained area in the aSyn + PREP injected PREPko group (n = 5). (**E,F**) Ratio between PK resistant oligomers or p129-aSyn stained particles and total aSyn oligomer count showed reduction in aSyn + PREP PREPko animal group (n = 4–6). (**G**) Representative images of aSyn and PK resistant oligomers and p129-aSyn staining in SN brain sections. Particularly in PREPko animals with aSyn + PREP injection, more diffuse staining in total aSyn, PK resistant aSyn oligomers and p129-aSyn is seen. Scale bar 5 μm. Bars represent mean ± SEM, 2-way ANOVA.

**Figure 3:**
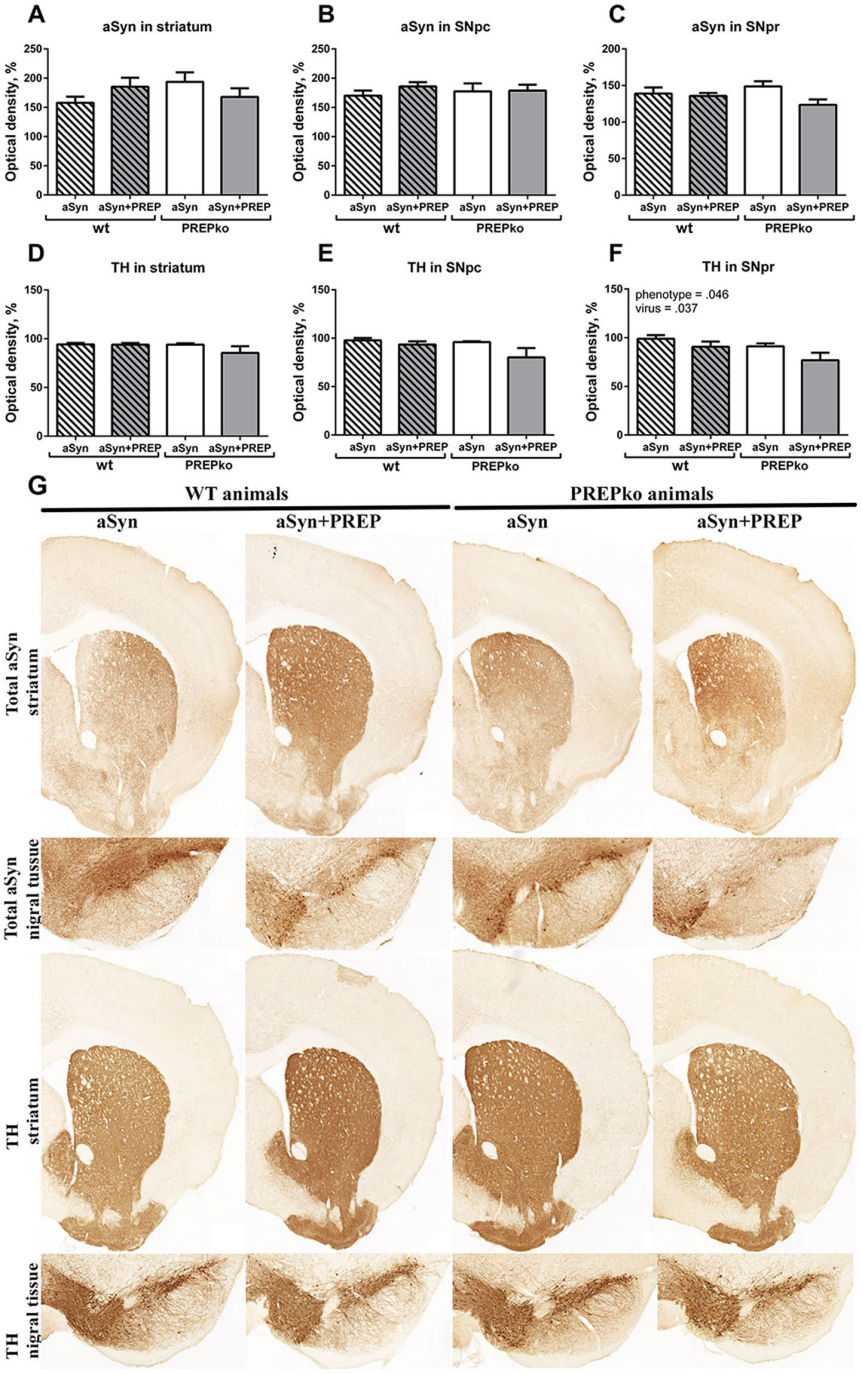
TH and total aSyn OD analyses showed minor changes in striatum and SN. (**A,B**) aSyn OD analyses in striatum and SNpc were not changed in any of the animal groups. (**C**) aSyn OD was not significantly altered in SNpr, however, a PREPko animals had a decreasing trend in aSyn after aSyn+ PREP injection. (**D,E**) TH+ fibers in striatum and SNpc were not changed in any of the animal groups. (**F**) SNpr OD analyses showed minor decrease of TH+ fiber density in aSyn+ PREP injected PREPko animal group that was in line with TH+ cell count (Fig. 2A). (**G)** Representative brain sections from striatal and nigral brain areas. n = 7–8. Bars represent mean ± SEM, 2-way ANOVA.

To determine whether PREPko animals after aSyn and aSyn + PREP injection exhibit similar aSyn staining pattern as wt animals, an oligomer specific aSyn staining was performed. Statistical differences were not observed in total aSyn oligomer specific staining using stereological investigation (Fig. 2B). We have previously reported that PREP inhibition by a small-molecule compound can reduce soluble oligomer amounts^15,17,18^ but the same observation did not hold true when PREP was knocked down. Proteinase K (PK) treatment was performed followed by aSyn oligomer specific staining and stereology. Statistical differences were observed between phenotype and viral vector injections (Fig. 2C; F_(1, 16)_ = 6.413, p = 0.022, 2-way ANOVA). Interestingly, aSyn + PREP injected PREPko mice had less PK resistant aSyn oligomers than aSyn injected PREPko animals, while the effect was not observed in wt animals (Fig. 2C). Visually, aSyn + PREP injected PREPko animals had lighter and more diffuse PK resistant aSyn oligomers in stereo-investigated SN brain sections (Fig. 2G). The ratio between the number of PK resistant aSyn particles and total amount of aSyn oligomer specific particles was matched for individual animals. Statistical interaction was observed between viral vectors and animal phenotype (Fig. 2E; F_(1,16)_ = 5.847, p = 0.028, 2-way ANOVA). PK resistant and total aSyn oligomer ratio in the PREPko animal group with aSyn + PREP injection was decreased while the opposite effect was seen between wt animal groups. Additionally, differences were not seen after optical density (OD) analyses of total aSyn IHC staining in striatum, *substantia nigra pars compacta* (SNpc) or *substantia nigra pars reticulate* (SNpr) (Fig. 3A–C,G).

### aSyn phosphorylation at serine 129 in SN

aSyn phospho S129 (p129-aSyn) was quantified to assess the distribution pattern differences between the phenotypes, additionally p129-aSyn particles were correlated with the total aSyn oligomer numbers in SN. We observed an interaction between the phenotype and viral vector injections for p129-aSyn phosphorylation (Fig. 2D,G; F_(1,16)_ = 8.657, p = 0.0096, 2-way ANOVA). The PREPko animal group with aSyn + PREP injections had the smallest area of the p129-aSyn staining (Fig. 2D) and it was visually more diffuse (Fig. 2G). The ratio between the number of particles and total amount of aSyn oligomer specific particles was matched for individual animals. No differences were observed between the groups (Fig. 2F).

### aSyn viral vector causes mild loss of nigrostriatal tyrosine hydroxylase

To study whether overexpression of aSyn + PREP affects DAergic neuron loss in mice differently from single aSyn microinjection, we performed stereological tyrosine hydroxylase positive (TH+) neuron quantification in SNpc and OD for TH+ fibers in SNpc, SNpr and striatum by IHC. The interaction effect between viral vectors and animal phenotypes was not statistically significant. Therefore, an analysis of the main effect for viral vector injection was performed, which indicated that the main effect between aSyn and aSyn+ PREP injection conditions was statistically significant (Fig. 2A; F_(1,23)_ = 7.965, p = 0.0097, 2-way ANOVA). TH+ cell loss was more pronounced in both animal groups that were injected with aSyn+ PREP. Our previous data has showed that PREP inhibition is partially protective against aSyn-caused toxicity^18^. Tyrosine hydroxylase (TH) OD analyses did not show a clear loss of TH+ fibers in either the striatum or the SNpc (Fig. 3D–E,G). However, in SNpr main effects for viral vector injection (Fig. 3F; F_(1,25)_ = 4.838, p = 0.037, 2-way ANOVA) and animal phenotype (Fig. 3C; F_(1,25)_ = 4.410, p = 0.046, 2-way ANOVA) were observed.

### No-net-flux microdialysis

**Figure 4:**
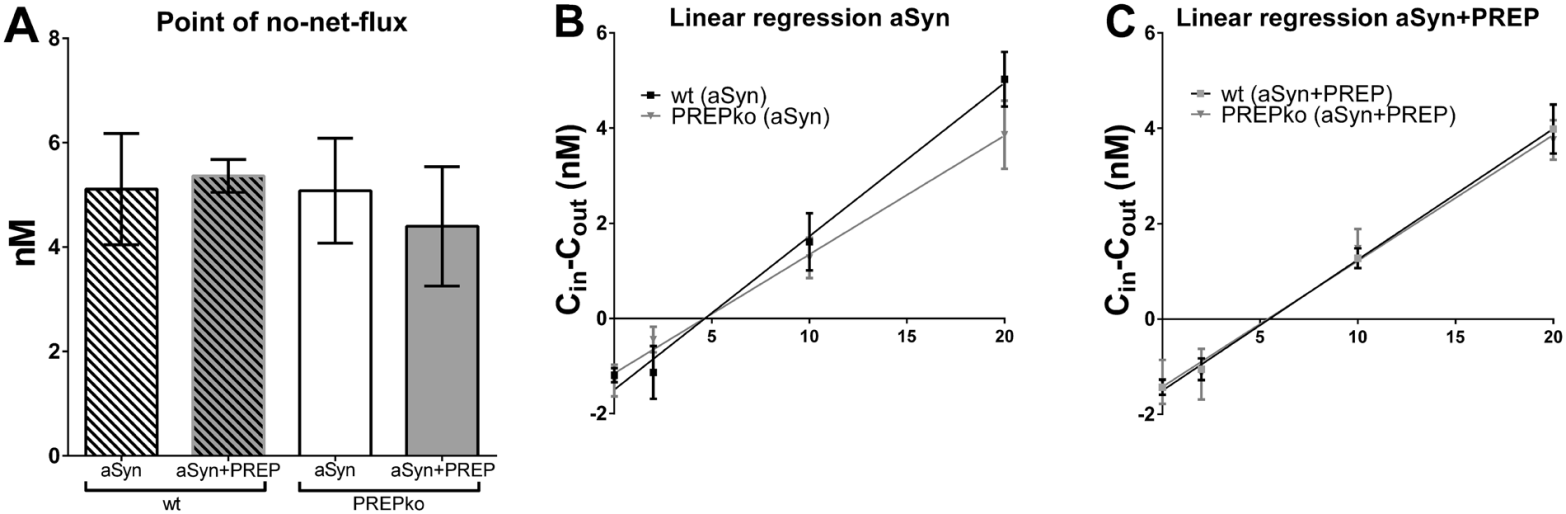
Extracellular DA levels in wt and PREPko mice after supranigral injection of aSyn or aSyn + PREP. (**A**) Extracellular DA level in the striatum was measured 14–15 week post-injection by no-net-flux microdialysis. Point of no-net-flux was similar in all groups but (**B**), there was a trend for a different extraction fraction (slope of the linear regression line) of the aSyn injected wt mice compared to that of the aSyn injected PREPko mice. (**C**) The extraction fractions were similar in wt and PREPko mice that received co-injection of aSyn + PREP (n = 7–9). Bars represent mean ± SEM.

The effect of aSyn and PREP overexpression on extracellular DA level was studied by striatal no-net-flux microdialysis 14–15 weeks post-injection. The extracellular concentration of DA was similar in aSyn and aSyn + PREP injected wt and PREPko mice (Fig. 4A) but there was a trend for a different extraction fraction (slope of the linear regression line) in the aSyn injected wt mice compared to that of the aSyn injected PREPko mice (Fig. 4B, F_(1,55)_ = 2.9949, p = 0.08913) indicating increased uptake of DA in the aSyn injected wt mice. The extraction fractions were similar in wt and PREPko mice that received co-injection of aSyn + PREP (Fig. 4C).

### HPLC tissue analysis

**Figure 5:**
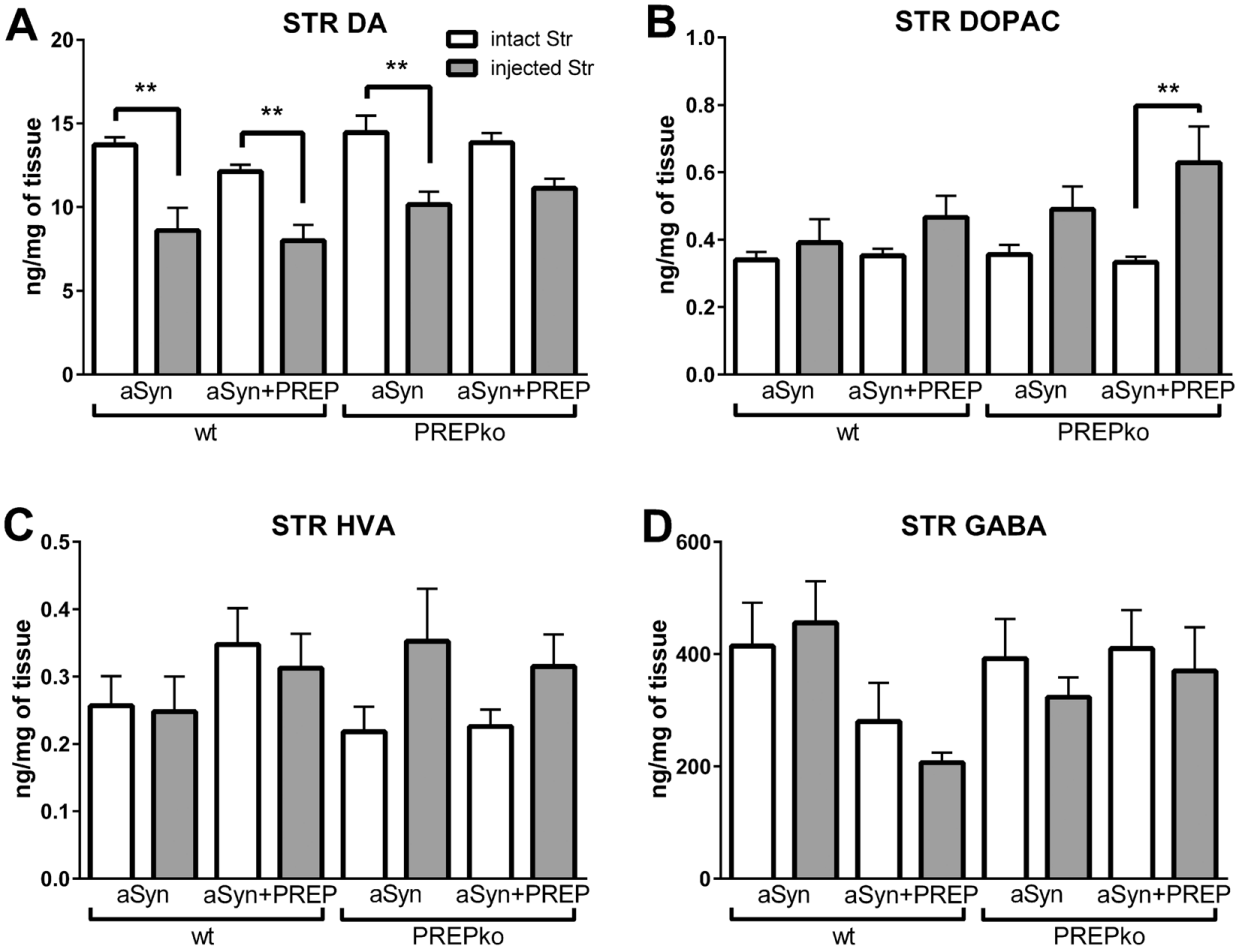
DA and DOPAC levels in aSyn + PREP injected PREPko mice is altered compared to other groups. (**A**) Tissue concentrations of neurotransmitters and their metabolites were measured 14–15 weeks post-injection by tissue HPLC analysis. Striatal DA was decreased in the injected striatum compared to the intact striatum in all groups except PREPko mice that received co-injection of aSyn + PREP but (**B**), the striatal DOPAC was increased only in aforementioned group. (**A**) The concentration of DA, (**B**) its metabolites DOPAC, (**C**) HVA and (**D**), GABA were similar between the groups. n = 7–9. Bars represent mean ± SEM. **p < 0.01, 1-way ANOVA with Tukey’s post hoc comparison.

To study whether the behavioral changes are caused by the nigrostriatal neurotransmitters, the tissue concentration of neurotransmitters and their metabolites were measured by tissue HPLC analysis. Concentration of DA (Fig. 5A), 3,4-Dihydroxyphenylacetic acid (DOPAC; Fig. 5B), homovanillic acid (HVA; Fig 5C), gamma-aminobutyric acid (GABA; Fig. 5D), 5-hydroxytryptamine (5-HT; Fig. S4) and 5-hydroxyindoleacetic acid (5-HIAA; Fig. S4) in the striatal tissue were similar in all groups. Striatal DA was significantly decreased compared to the intact side of the brain in all other groups (F _(7,56)_ = 9.403, wt-aSyn p = 0.0018, wt-aSyn + PREP p = 0.0082, PREPko-aSyn p = 0.0071, 1-way ANOVA with Tukey’s post hoc comparison) except in PREPko mice that received aSyn + PREP injection (F _(7,56)_ = 9.403, p = 0.2176), but DOPAC was increased significantly only in this group (F _(7,56)_ = 3.296, p = 0.0085).

### aSyn distribution from HEK-293 and PREPko cells in soluble, membrane bound and insoluble fractions

**Figure 6:**
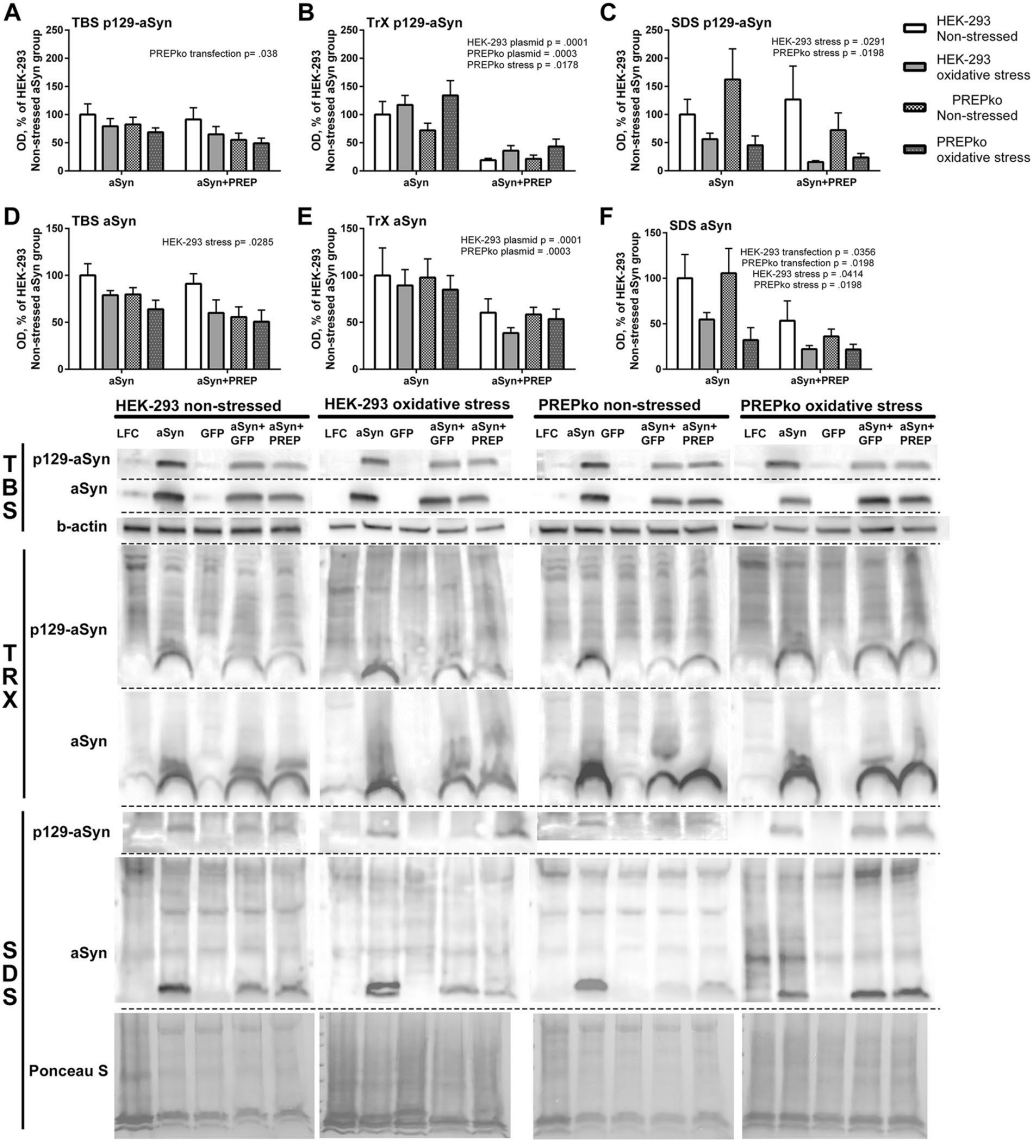
aSyn distribution between soluble (TBS), membrane bound (TrX) and insoluble (SDS) fractions in the presence of PREP overexpression. HEK-293 and PREPko cells were transfected with aSyn or aSyn + PREP and treated with oxidative stress for 48 hrs. Analyses of aSyn + GFP was not performed as the combination was very toxic to the cells but has been included in the WB images. (**A**–**C**), p129-aSyn levels significantly correlated with plasmid transfection and stress in PREPko and HEK-293 cells. (**A**) In TBS fraction, p129-aSyn was reduced in PREPko cells after aSyn + PREP transection. (**B**) Interestingly, combination of aSyn + PREP reduced considerably the levels of TrX p129-aSyn. (**C**) Moreover, oxidative stress significantly reduced the levels of SDS soluble p129-aSyn in both cell lines. (**D**–**F**), similar changes were seen in total aSyn levels, (**E**) although the decrease in TrX aSyn after aSyn + PREP transfection was not as notable as in p129-aSyn. (**F**)  Moreover, in SDS soluble aSyn, there was a significant reduction after oxidative stress in both cell lines, and also aSyn + PREP transfection lowered the SDS-aSyn expression. Full-length blots are presented in Supplementary Figure S1. Bars represent mean ± SEM, 2-way ANOVA.

Human embryonic kidney cells (HEK-293) and PREPko cells were used to establish aSyn distribution pattern in HEK-293 and PREPko cells after aSyn overexpression in the presence and absence of PREP and oxidative stress. Cells were lysed in 3 different fractions in order to distribute aSyn between its soluble, membrane bound and insoluble forms (see Fig. 6A–F). In TBS fractions, a moderate difference between aSyn and aSyn + PREP transfections could be observed on p129-aSyn levels in PREPko cells where p129-aSyn levels were slightly lower (Fig. 6A; F_(1,20)_ = 4.937, p = 0.038, 2-way ANOVA). Membrane bound fractions revealed a significantly reduced p129-aSyn levels after aSyn + PREP transfection both in HEK-293 (Fig. 6B; F_(1,20)_ = 28.99, p < 0.0001) and PREPko cells (Fig. 6B; F_(1,20)_ = 18.76, p = 0.0003). Additionally in PREPko cells, a significant increase in p129-aSyn levels was observed after oxidative stress (Fig. 6B; F_(1,20)_ = 6.668, p = 0.0178). Interestingly, insoluble fraction p129-aSyn levels were significantly decreased by stress in HEK-293 (Fig. 6C; F_(1,20)_ = 5.527, p = 0.0291) and PREPko cells (Fig. 6C; F_(1,20)_ = 6.410, p < 0.0198).

In the soluble fraction, oxidative stress significantly decreased the aSyn in HEK-293 cells (Fig. 6D; F_(1,20)_ = 5.571, p = 0.029). Membrane bound aSyn fractions were lowered in aSyn + PREP group both in HEK-293 (Fig. 6E; F_(1,20)_ = 5.839, p = 0.0254, 2-way ANOVA) and PREPko cells (Fig. 6E; F_(1,20)_ = 6.297, p = 0.0208). Surprisingly, the insoluble fraction of aSyn showed significantly decreased levels after oxidative stress (Fig. 6F; F_(1,20)_ = 4.750, p = 0.0414) and aSyn + PREP transfection reduced insoluble aSyn levels even further (Fig. 6F; F_(1,20)_ = 5.080, p = 0.0356) in HEK-293 cells. A similar effect was seen in PREPko cells (Fig. 6F; stress effect F_(1,20)_ = 7.461, p = 0.0129, plasmid effect F_(1,20)_ = 6.173, p = 0.0219).

**Figure 7:**
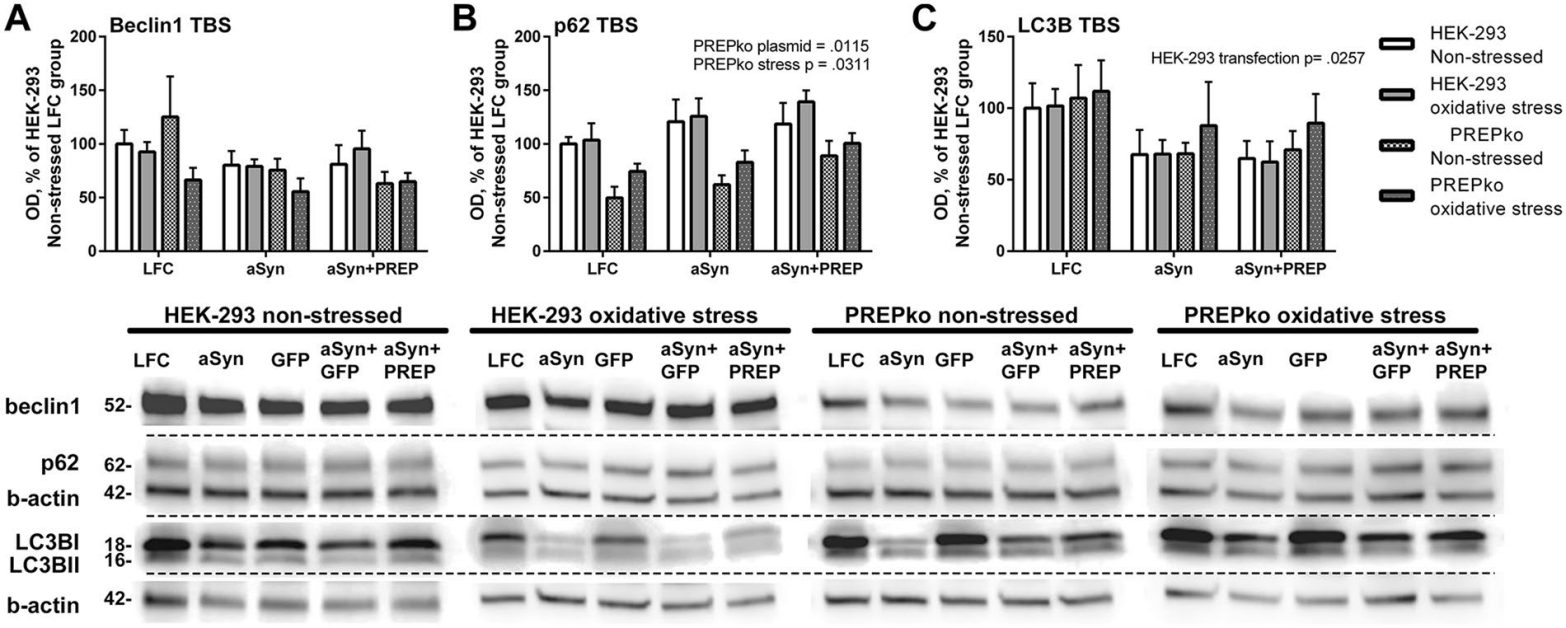
Autophagy markers in HEK-293 and PREPko cell soluble fraction (TBS) after transfection with aSyn or aSyn + PREP and treatment with oxidative stress for 48 hrs show slight alterations. Analyses of aSyn + GFP was not performed as the combination was very toxic to the cells but has been included in the WB images. (**A**) No effect was observed in beclin1 levels between cell lines or treatments. (**B**) p62 levels were lower in PREPko cells compared to HEK-293 cells, and stress and plasmid transfection significantly increased the p62 levels. The highest expression of p62 in PREPko cells was seen after combination of aSyn + PREP. (**C**) slight decrease of LC3BII levels was seen after transfections in both cell lines but it was significant only in HEK-293 cells. Full-length blots are presented in Supplementary Figure S2. Bars represent mean ± SEM, 2-way ANOVA.

Changes in autophagy markers were measured from the soluble fraction (Fig. 7) to establish if redistribution of aSyn and p129-aSyn is mediated via an autophagy pathway. Levels of p62, a protein accumulation marker, were significantly elevated by stress (Fig. 7B; F_(1,30)_ = 5.117, p = 0.0311, 2-way ANOVA) and transfections (Fig. 7B; F_(1, 30)_ = 5.203, p = 0.0115) in PREPko cells. However, the BL levels of p62 were approximately 50% lower in PREPko cells compared to HEK-293 cells (Fig. 7B). aSyn caused an increased accumulation of p62 compared to control treatment while the level of p62 was even greater than in the aforementioned groups, both non-stressed and stressed groups (Fig. 7B). Changes in beclin1 levels were not seen in either HEK-293 or PREPko groups (Fig. 7A). LC3BII levels were only mildly affected by aSyn and aSyn + PREP transfections in HEK-293 cells (Fig. 7C; F_(1,30)_ = 4.149, p = 0.0257).

### MTT cell viability assay

**Figure 8:**
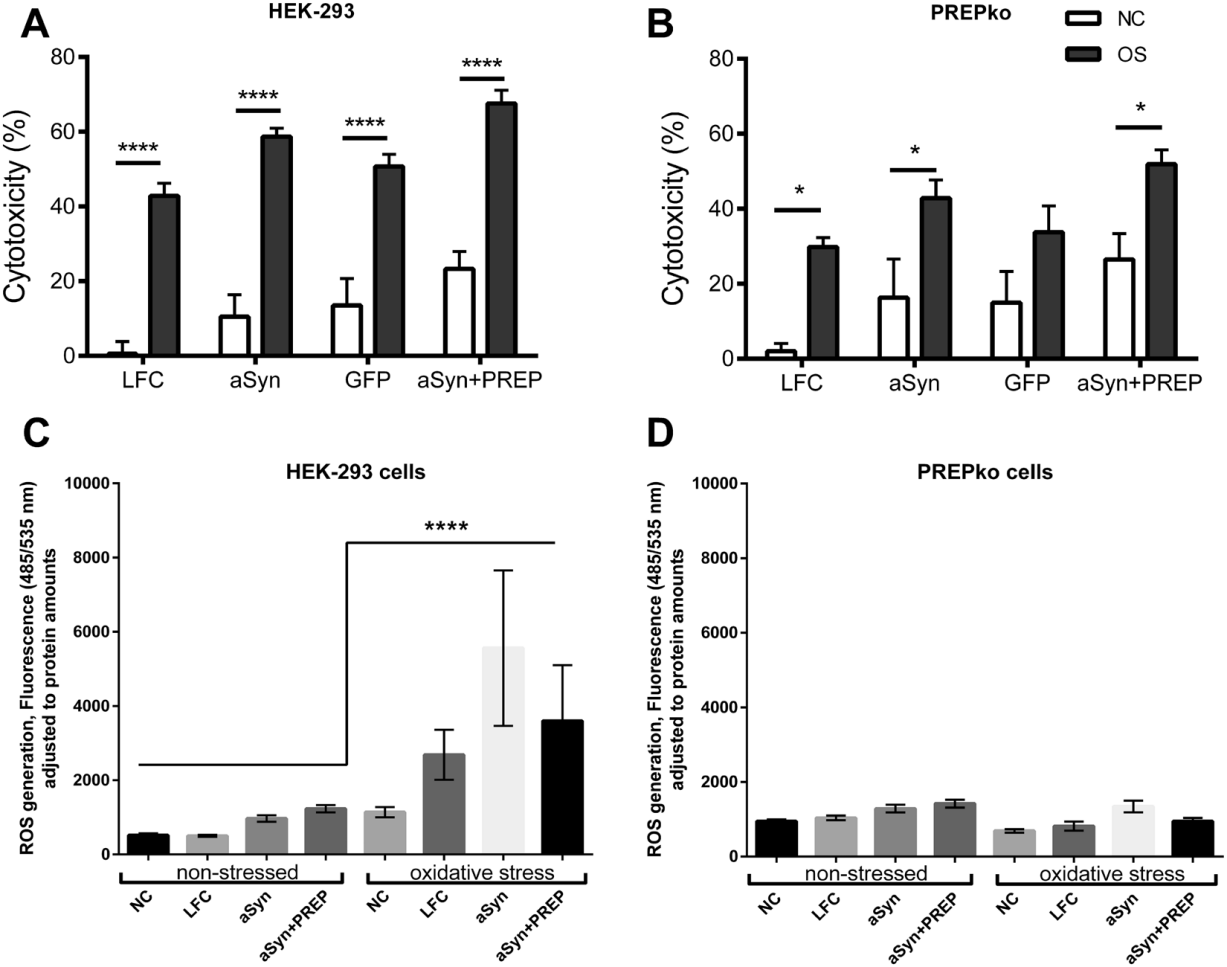
MTT cytotoxicity is the most pronounced in aSyn + PREP transfected cells. (**A**) There was a significant stress dependent effect in all HEK-293 oxidative stress (OS) groups. The aSyn + PREP transfected groups showed the highest cytotoxicity while the aSyn transfected cell group had significantly reduced cell numbers only compared to control group. (**B**) Oxidative stress (OS) effect in PREPko cells was less pronounced, and only in aSyn + PREP transfected group increased cytotoxicity to control groups were seen. 2-way ANOVA with Tukeys’s post hoc comparison. (**C**) ROS were statistically increased in the HEK-293 cells after oxidative stress treatment. (**D**) ROS level alterations between non-stressed and oxidative stress PREPko groups were not observed. Univariate analyses of variance with Bonferroni’s adjustment. Bars represent mean ± SEM. *p < 0.05, **p < 0.01, ****p < 0.0005.

A cell specific response to either aSyn or aSyn + PREP plasmid overexpression in the presence of oxidative stress medium was followed by MTT assay to establish a cell type specific response to the oxidative stress. A cytotoxicity by oxidative stress was observed among all HEK-293 groups (Fig. 8A; F_(1,23)_ = 183.9, p < 0.0005, 2-way ANOVA) and PREPko groups (Fig. 8B; F_(1,23)_ = 28.10, p < 0.0005). Although all transfections had an effect on cell viability in HEK-293 cells (Fig. 8A; F_(1,23)_ = 28.10, p < 0.0005), aSyn + PREP group and all other groups (p = 0.025 vs. aSyn, p = 0.007 vs. GFP and p < 0.0005 vs control group) as well as between control group and aSyn group (p = 0.035). The plasmid transfection effect in PREPko cells was milder and observed only between negative control and aSyn + PREP groups (p = 0.02) (Fig. 8B; F_(3,23)_ = 4.271, p = 0.015, 2-way ANOVA with Tukey’s post hoc comparison). Overall cell death in PREPko cells exposed to stress was lower (Fig. 8B).

### Cellular reactive oxygen species production and oxidative stress defense marker upregulation in the response of oxidative stress

**Figure 9:**
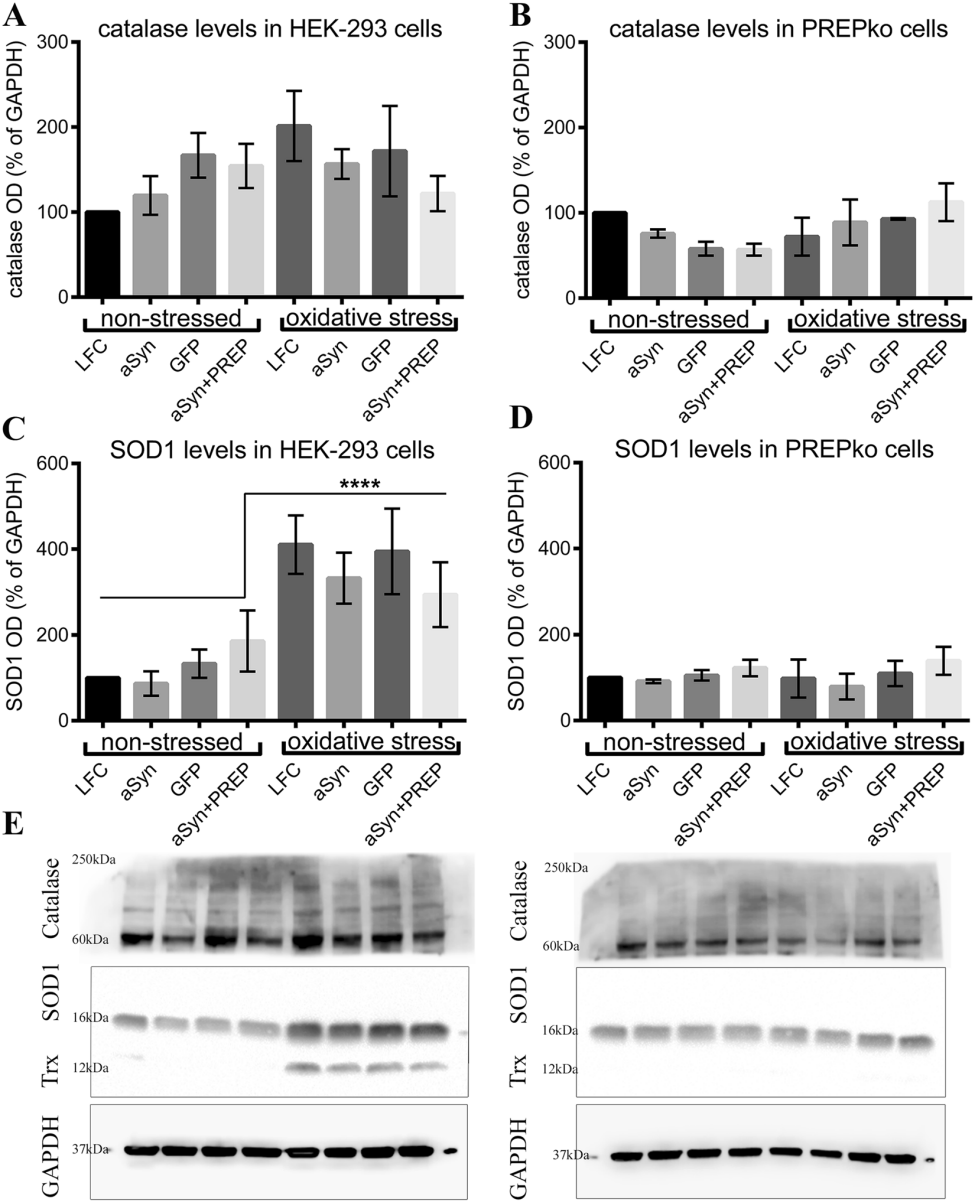
PREPko cells does not respond to oxidative stress. (**A,B**) Catalase activity in the HEK-293 and PREPko cells did not show alterations. (**C**) Superoxide dismutase 1 (SOD1) was statistically increased in the HEK-293 oxidative stress groups compared to the non-stressed HEK-293 group. (**D**) No differences in SOD1 levels were detected between PREPko groups. (**E**) Representative images of WB bands for catalase, SOD1 and thioredoxin (Trx) in HEK-293 and PREPko cells in the presence and absence of oxidative stress. Bars represent mean ± SEM. ****p < 0.0005. Univariate analyses of variance with Bonferroni’s adjustment.

Reactive oxygen species (ROS) were measured in HEK-293 and PREPko cells using 2′, 7′-Dichlorofluorescin diacetate (DCFDA). The fluorescence was significantly increased in HEK-293 oxidative stress groups compared to non-stressed HEK-293 (Fig. 8C; F_(1,172)_ = 31.26, p < 0.0005) and PREPko oxidative stress group (Fig. 8D; F_(1,172)_ = 27.38, p < 0.0005). To our surprise ROS production differences were not observed between PREPko groups (two-way interaction for stress*phenotype F_(1,172)_ = 18.85, p = 0.000032, Univariate analyses of variance with Bonferroni’s adjustment). Similarly, Western blot (WB) was used to measure catalase, superoxide dismutase 1 (SOD1) and thioredoxin levels in HEK-293 and PREPko cell TBS fractions in the presence and absence of oxidative stress. SOD1 were statistically increased in the HEK-293 oxidative stress groups compared to the non-stressed HEK-293 (Fig. 9C,E; F_(1,32)_ = 47.45, p < 0.0005) and PREPko oxidative stress group (Fig. 9C–E; F_(1,32)_ = 55.95, p < 0.0005). Similar to ROS levels, we did not observe increased levels of SOD1 or thioredoxin in PREPko cells in the presence of oxidative stress (Fig. 9D,E; two-way interaction for stress*phenotype F_(1,32)_ = 23.40, p = 0.000032, Univariate analyses of variance with Bonferroni’s adjustment). We did not observe changes in catalase activity between groups (Fig. 9A,B,E) while thioredoxin levels were not analyzed as marker was visible only in the HEK-293 oxidative stress group, indicating that PREPko cells do not upregulate enzyme in particular oxidative stress condition (Fig. 9E).

### Proteasomal activity alterations in PREPko cells

**Figure 10:**
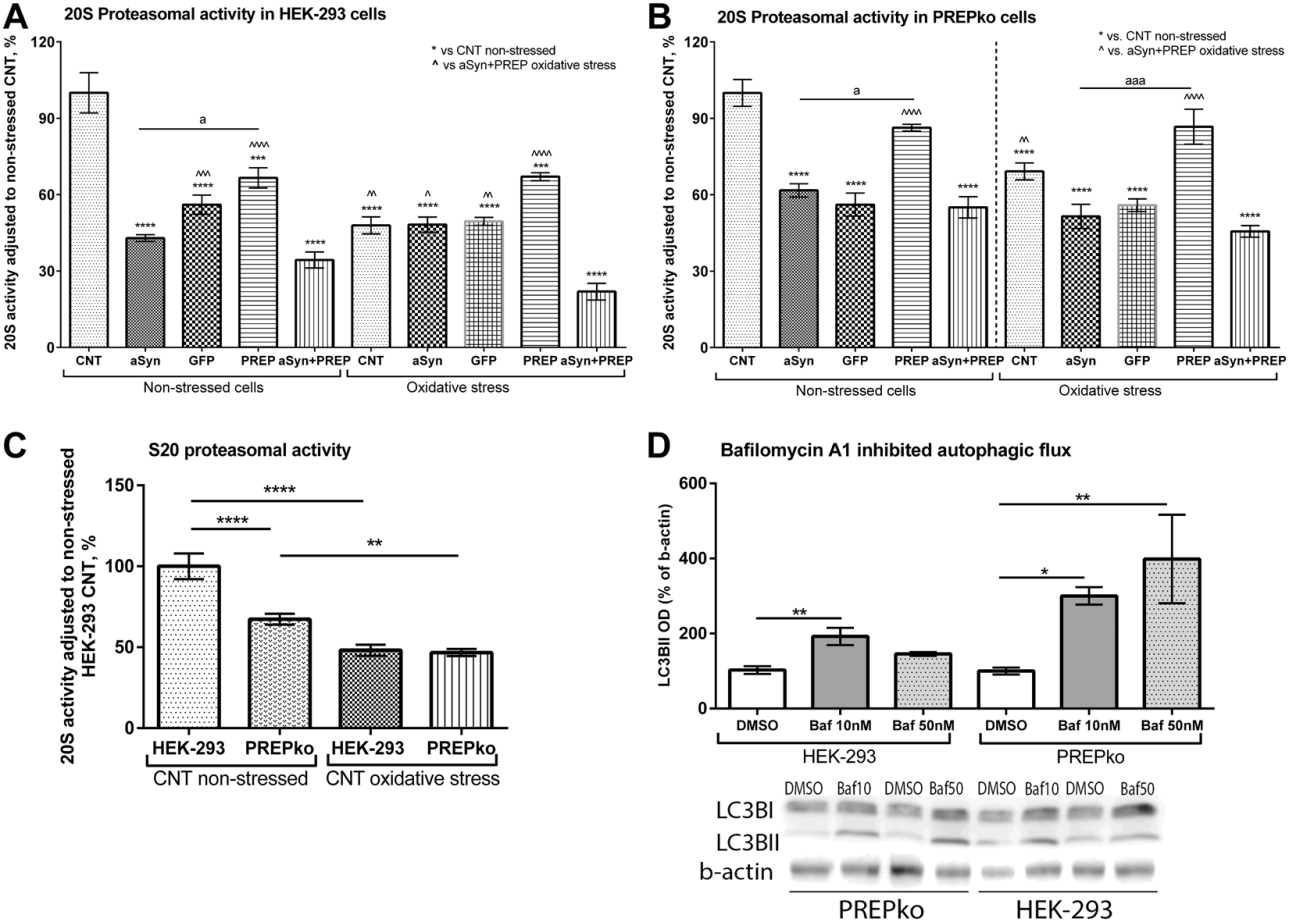
HEK-293 and PREPko cells showed a different proteasomal activity response to the aSyn + PREP protein overexpression. (**A**) HEK-293 cells transfected with aSyn + PREP showed the largest decrease in the proteasomal activity after oxidative stress addition while in non-stressed conditions aSyn and aSyn + PREP groups were not statistically different (2-way ANOVA with Tukey’s post hoc comparison). (**B**) PREPko cells were less responsive to proteasomal activity decrease in stress conditions and both aSyn and aSyn + PREP transfected cells behaved similarly (2-way ANOVA with Tukey’s post hoc comparison). (**C**) Basal S20 proteasomal activity was decreased in PREPko cells while no differences were observed between cell types in the presence of oxidative stress (2-way ANOVA). (**D**) PREPko cells showed increased LC3BII accumulation after bafilomycin A1 (Baf) treatment, pointing to increased autophagic flux. 4 hr incubation with Baf 10 nM concentration showed increased autophagosome accumulation as assessed by LB3BII in HEK-293 cells but in PREPko cells there was significantly elevated LC3BII levels after 10 nM and 50 nM bafilomycin A1 inhibition (1-way ANOVA with Tukey’s post hoc comparison). Full-length blots are presented in Supplementary Figure S3. Bars represent mean ± SEM. ^a,^*^,^^p < 0.05, **^,^^^p < 0.01, ***^,^^^^^,aaa^p < 0.001, ****^,^^^^^p < 0.0005.

Proteasomal activity was measured in HEK-293 and PREPko cells in the presence and absence of oxidative stress. In HEK-293 cells, both oxidative stress and plasmids transfections reduced the proteasomal activity (Fig. 10A; F_(4,36)_ = 16.58, p < 0.0001, 2-way ANOVA with Tukey’s post hoc comparison). Non-stressed control group’s S20 proteasomal activity was statistically higher compared to the rest of the groups (see Fig. 10A). The oxidative stress conditioned aSyn + PREP group had the most decreased proteasomal S20 activity in HEK-293 cells (Fig. 10A), where it was lower compared to the rest of the oxidative stress groups (vs. control group (p = 0.0029) and GFP group (p = 0.008); vs. aSyn group (p = 0.013)). While in non-stressed conditions, the aSyn and aSyn + PREP group proteasomal activity was not statistically different (Fig. 10A). An important observation is the statistical difference between HEK-293 cell aSyn and PREP transfected group in non-stressed conditions (p = 0.037) while a difference is not observed with oxidative conditions.

PREPko cells have decreased proteasomal activity compared to the HEK-293 cells at the BL level (p < 0.0005) while differences between cell types were not observed in the presence of oxidative stress (Fig. 10C; F_(1,28)_ = 12.842, p < 0.001, 2-way ANOVA), which could be explained partially due to increased autophagic flux in PREPko cells (See Fig. 10D). Nevertheless, similar to HEK-293 cells, PREPko cells had a significant 2-way interaction between stress and plasmid transfection conditions (Fig. 10B; F_(4,36)_ = 5.480, p = 0.0015, 2-way ANOVA with Tukey’s post hoc comparison). When post hoc comparison was done for PREPko cell 20S proteasomal activity, the non-stressed control group had statistically higher 20S proteasomal activity compared to all the groups except the PREP transfected non-stressed and oxidative stress conditions (Fig. 10B). The aSyn + PREP oxidative stress groups were statistically different only to non-stressed control group and PREP transfected group (p < 0.0005) and oxidative stress control (p = 0.005) and PREP transfected group (p < 0.0005). Moreover, only PREP transfected groups did not show statistical differences to both control groups (Fig. 10B). When aSyn and PREP transfected groups were compared, a statistical difference was observed between the non-stressed aSyn and PREP groups (p = 0.015) and oxidative stress aSyn and PREP group (p = 0.0001), that indicated that in PREPko cells overexpression of PREP alone does not significantly decrease 20S proteasomal activity.

### Autophagic flux alterations in PREPko cells

Accumulation of autophagosomes was observed in HEK-293 and PREPko cell lines after 4 hr 10 nM and 50 nM bafilomycin A1 treatment (Fig. 10D). HEK-293 cells showed a statistically significant increase of autophagosomes (F_(2,9)_ = 11.02, p = 0.0038, 1-way ANOVA with Tukey’s post hoc comparison), while concentration dependent accumulation of autophagosomes was seen in PREPko cells (F_(2,9)_ = 10.15, p = 0.0038). HEK-293 cells at 10 nM had an approximately 2-fold increase (p = 0.0031) while PREPko cells had a 3-fold increase at 10 nM (p = 0.046) and 4-fold increase at 50 nM (p = 0.0055) concentration of bafilomycin A1 compared to respective control, suggesting that PREPko cells have increased autophagic flux (Fig. 10D).

### aSyn levels in cell medium are increased in PREPko cells transfected with aSyn plasmid

**Figure 11:**
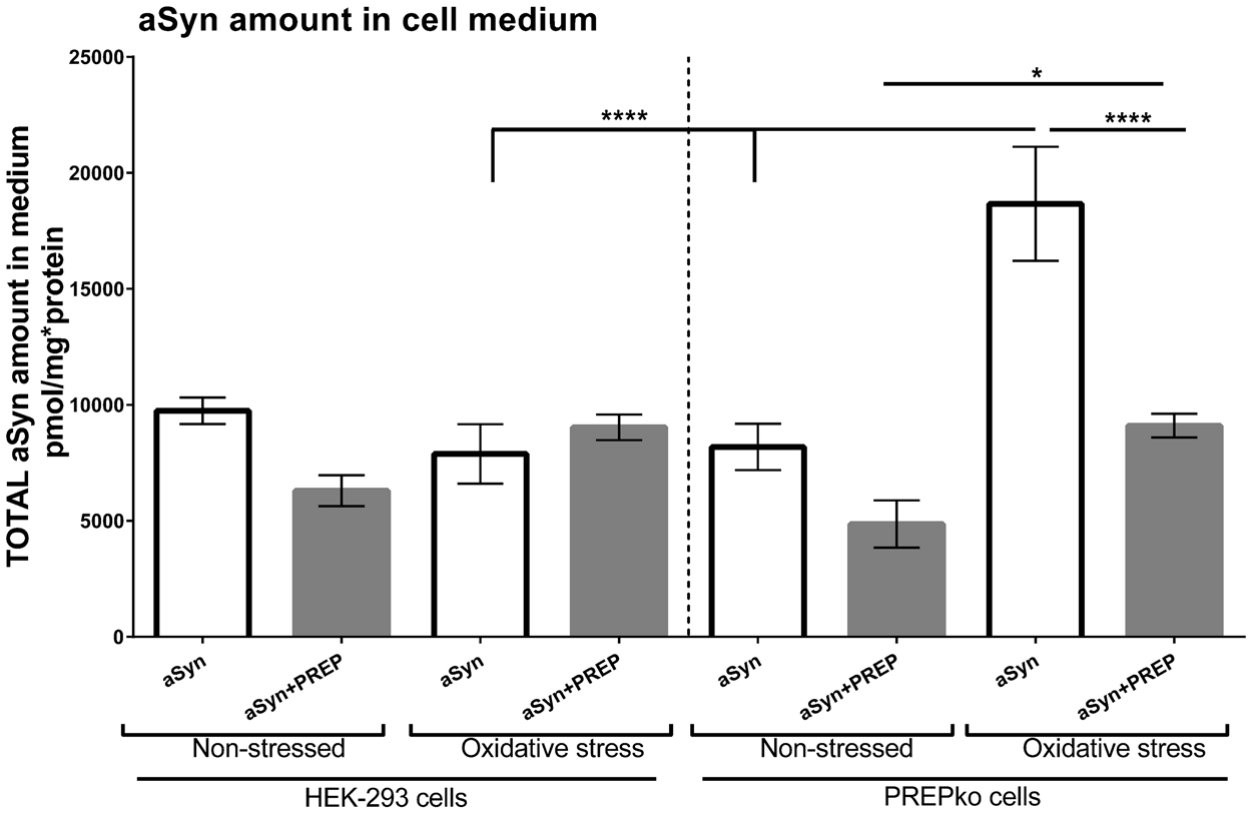
PREPko cells in stress condition have increased levels of extracellular aSyn only in the aSyn transfected group of PREPko cells compared to non-stressed PREPko cells or stressed HEK-293 cells with aSyn transfection. Interestingly, restoring PREP to PREPko cells with aSyn reduced extracellular aSyn to control levels. Bars represent mean ± SEM, *p < 0.025, ****p < 0.0005, 3-way ANOVA.

We measured aSyn levels in cell medium after we noticed that some of the data from WB showed an apparent decrease of total aSyn in membrane bound and insoluble fractions. HEK-293 and PREPko cells were used in non-stressed and oxidative stressed conditions to measure amount of aSyn in cell medium. There was a significant increase in aSyn transfected PREPko cells after oxidative stress compared to HEK-293 cells (Fig. 11; p < 0.0005). PREPko cells showed a significant oxidative stress-related increase of secreted aSyn between aSyn transfected PREPko cells (p < 0.0005) and aSyn + PREP transfected PREPko cells (Fig. 11; p = 0.021). A significant interaction effect was observed between cell phenotype, stress, and plasmid transfection conditions (Fig. 11; F_(1, 16)_ = 10.622, p = 0.005, 3-way ANOVA). There was a statistically significant simple 2-way interaction between stress conditions and cell phenotype for aSyn treated groups (F_(1,16)_ = 27.577, p < 0.0005). Moreover, there was a statistically significant simple two-way interaction between cell phenotype and transfection conditions for oxidative stress groups (F_(1,16)_ = 20.755, p < 0.0005) but not for non-stressed groups.

Additionally, a cut off column with 30-kDa exclusion size was used to measure the monomeric and dimeric aSyn in the cell medium. However, the monomeric and dimeric aSyn levels were too low in the medium to be detected with ELISA and this proposes that most of the secreted aSyn in cell medium was higher order aSyn forms.

## Discussion

In this study, our aim was to clarify the significance of PREP for aSyn aggregation and toxicity *in vivo* and reveal the mechanisms by using cell culture models. We showed that PREPko animals were not sensitive for overexpression of aSyn by using unilateral viral vector injections in the nigrostriatal pathway and restoring PREP together with aSyn significantly reduced animal locomotor activity. Moreover, biochemical investigation revealed that aSyn modifications and TH+ cell loss in the PREPko animal phenotype injected with aSyn + PREP viral vectors was the most pronounced. Cell experiments corroborated the *in vivo* data since combination of aSyn + PREP transfection in PREPko cells was found to be the most toxic. Moreover, our cellular data explained in part the mechanisms behind the PREP mediated aSyn toxicity, notably the changes in aSyn clearance and phosphorylation.

PREPko animals that received aSyn + PREP injection exhibited significant changes in their locomotor behavior. Our group had previously tested the effects of PREP protein restoration in PREPko mice but significant changes were not observed^11^. Initial PREP protein detectability in striatum^11^ coincides with the period when the first divergence in the motor behavior between PREPko mouse groups appear. Consequently, it indicates that locomotor activity decrease is due to the synergistic toxic effect of aSyn and PREP overexpression rather than rescue of the PREPko animal phenotype. Additionally, only wt animals exhibited changes in the paw preference, similar to our previous study showing that the unilateral aSyn viral vector overexpression produces changes in paw preference as early as 2 weeks post-injection^18^. Interestingly, PREPko animals did not exhibit unilateral paw misbalance but it could be attributed to the increased extracellular levels of DA in the naïve PREPko animal striatum as we have shown in our earlier study^11^.

Overexpression of aSyn decreased striatal tissue concentration of DA to an average of 60–70% of the concentration in the intact side of the brain, however striatal DA was decreased less in aSyn + PREP injected PREPko mice. Restoring PREP to PREPko mice elevated striatal DA level in our previous study^11^, and the same effect can be seen to some extent in this study. Overexpression of aSyn induced a decrease in striatal DA as reported earlier by us^18^ and others^22–26^. In our previous study^11^, naïve PREPko mice had a higher extracellular level of DA than wt littermates but in this study, we did not find this difference between the injected hemispheres. aSyn regulates extracellular DA under normal conditions by stabilizing DAT on the plasma membrane^27^ and our previous study showed that PREPko mice have impaired DAT function and more internalized DAT^11^. Moreover, aSyn can alter the synaptic DA vesicle genesis and recycling that leads to decreased release of DA^19^. This indicates that the overexpression of aSyn regulates nigrostriatal DA and DAT function strongly enough to cover the effect of absence or restoration of PREP. Extracellular and tissue concentration of DA did not correlate with the behavioral changes most likely because both aSyn and PREP regulate the nigrostriatal DAergic system. Elevated DOPAC in the striatum of aSyn + PREP injected PREPko mice can indicate altered DA metabolism by monoamine oxidase and increase in more toxic metabolite DOPAL. Increased DOPAC and DOPAL could possibly increase oxidative stress, impair synaptic vesicle function and prevent aSyn fibrillation by stabilizing aSyn oligomers^28,29^.

PREPko mice that received aSyn + PREP injection had lower levels of phosphorylated aSyn and the particles were more diffuse. There are reports that aSyn phosphorylation, especially p129-aSyn modification, is decreasing neurotoxicity in the drosophila^30^ and rat models of PD^31^. Besides, p129-aSyn modification has been shown to facilitate degradation of aSyn via autophagic and proteasomal pathways^32^. The decreased levels of p129-aSyn that we observed in aSyn + PREP injected PREPko animals could be due to post-translational modifications that reduce aSyn degradation, especially as p129-aSyn modification is shown to decrease aSyn half-life^32^. Additionally, aSyn + PREP injected PREPko animals had a lower amount of PK resistant aSyn oligomers. It has been shown that PK resistant oligomers and Lewy body structures are part of the cell’s coping mechanism that reduces cell exposure to the toxic aSyn species^33–35^. Besides, SNpc neurons that do not contain Lewy bodies could represent a neuronal survival strategy^36,37^. Nevertheless, TH+ stereology in SNpc indicated a slightly higher cell loss in the aSyn + PREP injected animal groups. All animal groups apart from the PREPko animals injected with aSyn + PREP showed clear aSyn inclusion bodies. PREP has been shown to increase aSyn dimerization in cell free conditions and via direct protein-protein interaction^16^ while in mouse models, inhibition of PREP activity reduced aSyn amount in a transgenic mouse strain^17^ and in the viral vector overexpression model of PD^18^. However, some of the observed IHC differences between animal phenotypes could be due to the PREPko animal signaling pathway alterations^11^. The ability of the PREP protein to increase aSyn dimerization could lead to a faster accumulation of soluble, more toxic aSyn species^33^ that could not be detected with stereology.

Similar to *in vivo* results, PREPko cells were more resistant to the toxicity of aSyn plasmid transfection and oxidative stress, and in both cell lines, the combination of aSyn + PREP transfection was the most cytotoxic. Although oxidative stress is commonly used to initiate aSyn aggregation, we did not see a significant elevation of Triton X-100 or sodium dodecyl sulfate (SDS) soluble aSyn particles after oxidative stress apart from membrane-bound S129 phosphorylated aSyn, but aSyn particles were decreased after stress, and this was seen both in HEK-293 and PREPko cells and after aSyn + PREP transfections as well. Surprisingly, when we studied the oxidative stress response in cells, we found that ROS levels and ROS response protein SOD1 and thioredoxin levels were elevated in the HEK-293 cells after oxidative stress induction but no alterations were seen in PREPko cells. Catalase levels in HEK-293 and PREPko cells were similar, indicating that PREP role in some of the oxidative stress response is likely upstream of the hydrogen peroxide (H_2_O_2_) reduction^38^. Additionally, the PREPko cell apparent lack of SOD1 upregulation could indicate that these cells does not produce as much superoxide^39^ or that PREPko cells would reduce H_2_O_2_ via thioredoxin system^40^. Another possibility is that deletion of PREP could reduce excessive H_2_O_2_ via other redox or autophagy pathways^41^. However, further studies are required to establish mechanisms that govern the cell survival upon PREP deletion.

We found that cells with aSyn + PREP plasmid transfection inhibited the most proteasomal activity that is indicative of the decreased aSyn protein degradation^42,43^, and PREP inhibition has been shown to attenuate toxicity of proteasomal inhibition in aSyn overexpressing cells^44^. In this study, PREPko cells had lower basal 20S proteasomal activity in comparison to the control cells. However, PREPko cell proteasomal activity changes in the presence of oxidative stress and protein overexpression were lower. Some evidence suggests that low-level proteasomal inhibition could have a beneficial effect on neuroprotection^37,42,43,45^. The proteasomal system is the main aSyn degradation pathway during basal conditions but with increased stress the autophagic pathway is recruited^43,46^. PREP is thought to act as a negative autophagy regulator^17^, and we saw an increased basal autophagic flux in PREPko cells. After co-transfection of aSyn and PREP, PREPko cells showed decreased proteasomal activity and as PREP is known to inhibit autophagic flux, it adds a subsequent strain on the autophagic-lysosome systems ability to cope with the aSyn overexpression.

Since we saw reduced levels of different aSyn forms after oxidative stress, we wanted to measure the aSyn secretion. PREPko cells with aSyn transfection had significantly increased extracellular aSyn levels and showed the largest difference between stressed and non-stressed conditions in SDS soluble fractions. PREP restoration reduced aSyn level in cell medium to the levels of control cells. It seems that PREPko cells are able to remove excessive aSyn by facilitating cytosolic aSyn transport. Despite that, PREP’s role in vesicular trafficking has not been shown apart from a report that PREP could be involved in axonal transport by an unknown mechanism^47^. It has been shown that aSyn can be secreted in the cell medium via a non-conventional vesicular pathway^48^ and exosomes^49,50^. Moreover, aSyn can be released in the extracellular space upon autophagic failure^51^ and lysosomal dysfunction^52^. Although extracellular aSyn can be potentially toxic, our results indicate that PREPko cells do not exhibit a strong cytotoxic response even with high amounts of aSyn in the extracellular space although most of the aSyn in cell medium were higher order aggregates.

Studies with PREP have shown beneficial effects on aSyn aggregation and clearance after PREP inhibitor treatment^16–18,44,53^. However, information about aSyn toxicity in the total absence of PREP has not been reported. We were able to show that co-overexpression of aSyn and PREP in PREPko animals and cells increases toxicity and ablates the ability of the proteasomal systems to process aSyn. Even though we could not identify the toxic aSyn species that presence of PREP causes, PREPko cells had several mechanisms to cope with the increased aSyn load, such as increased autophagy and increased aSyn oligomer transport into the cell medium. Taken together, although aSyn forms aggregates in the absence of PREP, our findings showed that PREP is important for aSyn-mediated toxicity and this further emphasizes the possibilities of PREP inhibitors as disease-modifying drugs for PD and other synucleinopathies.

## Materials and Methods

### Reagents

Reagents were purchased from Sigma-Aldrich if not otherwise specified. Ethanol was purchased from Altia (Helsinki, Finland). Adeno-associated virus (AAV) driven by chicken β-actin promoter (CBA) was acquired from the MichaelJ. Fox Foundation. AAV2-CBA-α-synuclein (AAV-aSyn; 1.5 × 10^13^ vg/mL) viral vectors were constructed, produced, and titered by Vector Core at the University of North Carolina (Chapel Hill, USA). AAV1-EF1α-PREP was obtained from the National Institute of Drug Abuse (Dr. Brandon Harvey, Intramural Research Program, Baltimore MD, USA). Plasmids, pAAV1-EF1α-PREP (Addgene #59967), AAV1-EF1α-GFP (Addgene #60058) and AAV1-EF1a-V5-synuclein (Addgene #60057) were obtained from National Institute of Drug Abuse (Dr. Brandon Harvey, Intramural Research Program, Baltimore, MD, USA). Plasmid construction was described in^17^ while PREP viral vector characterization can be found in^11^.

### Animals

PREPko mice (Deltagene Inc, CA, USA) and wt littermates were back crossed in C57BL/6JRccHsd genetic background (Envigo, The Netherlands; 5–10 back crossings). Generation of PREPko mice has been described in^54^, behavioral phenotyping was done by^13^. Mice (7–9 weeks old; Envigo, The Netherlands) were housed under standard laboratory conditions. The experiments were carried out according to the European Communities Council Directive 86/609/EEC and were approved by the Finnish National Animal Experiment Board.

### Surgical procedures

Mice were anesthetized with isoflurane (4% induction, 1.5–2.0% maintenance) and the recombinant AAV vectors were injected above mouse SN in a stereotaxic operation. AAV vectors were tested earlier^11,18^. To target the SN, viral vectors were given as single injection (volume 1 µL or 2 µL co-injection, rate 0.2 µL/min) into the left hemisphere, 3.1 mm anterior and 1.2 mm lateral to bregma, and 4.2 mm below the dura^55^.

Guide cannula (AT4.9.iC, AgnTho’s, Sweden) for microdialysis was inserted into the striatum at 0.6 mm anterior and 1.8 mm lateral, and 2.7 mm below the dura. The cannula was fastened to the skull with dental cement (Aqualox, Voco, Cuxhaven, Germany) and two stainless steel screws (1.2 × 3 mm, DIN84, Helsingin Ruuvihankinta, Finland).

### Availability of materials and data

The datasets generated during and/or analyzed during the current study are available from the corresponding author on reasonable request.

## Behavioral experiments

### Cylinder test

Cylinder test (height 15 cm, diameter 12 cm) was used to measure motor asymmetry in spontaneous forelimb preference after unilateral microinjections as described in^18^. Animals were grouped by their BL paw preference prior to the surgery. No habituation of the animals to the testing cylinder was allowed before video recording.

### Open field activity

The mice were released in the corner of an open field arena (30 × 30 cm, Med Associates, GA, USA) and activity was recorded at the same time of the day for all of the animals. Total distance (locomotor activity) and vertical activity were analyzed. Data was collected in 5 min intervals and activity was recorded for 120 min (light intensity 150 lx). Data was analyzed with Activity Monitor v 7.06 software.

### Tissue processing

At 13 weeks post-injection, mice intended for IHC analysis were deeply anesthetized with sodium pentobarbital (150 mg/kg) and transcardially perfused as described in^18^. Frozen brain sections were sectioned as 30 µm free-floating sections on a cryostat (Leica CM3050, Wetzlar, Germany) and kept in a cryoprotectant solution.

### Immunohistochemistry

Total aSyn IHC was performed as described in^15^. In brief, non-specific binding was blocked with 10% normal donkey serum (#S30, Millipore, Temecula, CA, USA). The sections were incubated overnight at room temperature with sheep anti-aSyn antibody (1:500; ab6162, RRID:AB_2192805, AbCam, Cambridge, UK). The sections were then incubated with donkey anti-sheep HRP conjugated secondary antibody for 2 h (1:500; ab6900, RRID:AB_955452, AbCam). The antigen–antibody complexes were identified following incubation with 0.05% 3,3′-diaminobenzidine (DAB) and 0.03% H_2_O_2_ solution. Finally, the sections were transferred to glass slides, dehydrated in alcohol series and mounted with Depex (BDH, Poole, UK).

TH IHC was modified from^56^. In short, sections were incubated for 30 min in 10% normal goat serum (S-1000, RRID:AB_2336615, Vector laboratories, Peterborough, UK). Afterwards sections were incubated overnight in rabbit anti-TH primary antibody (1:2000 dilution in 1% normal goat serum; AB152, RRID:AB_390204, Millipore). Subsequently, the sections were placed in goat anti-rabbit biotin conjugated secondary antibody (1:500 dilution in 1% normal goat). The signal was enhanced with the avidin–biotin complex-method (Standard Vectastain ABC kit, RRID:AB_2336819, Vector Laboratories) and visualized with DAB.

p129-aSyn staining was done as described above. Sections were incubated overnight in rabbit anti-pSer129alpha-synuclein primary antibody (1:250 dilution; ab59264, RRID:AB_2270761, AbCam). Subsequently, the sections were placed in anti-rabbit HRP secondary antibody (1:500; #31460, RRID: AB_228341, Thermo Fisher Scientific, Waltham, USA).

Oligomer specific aSyn IHC was performed as described in^18^. In short, the sections were incubated for 30 min in M.O.M. Mouse Ig Blocking Reagent to block nonspecific staining and 5 min in M.O.M. diluent (Basic Vector M.O.M. Immunodetection Kit, BMK-2202, RRID:AB_2336833, Vector laboratories). Sections were transferred overnight in mouse anti-human aSyn oligomer specific primary antibody (1:200; AS132718, RRID:AB_2629502, Agrisera, Sweden). The sections were then incubated with goat anti-mouse HRP conjugated secondary antibody (1:300, #31430, RRID:AB_228307, Thermo Fisher Scientific).

PREP IHC was previously described in^11^. In brief, non-specific binding was blocked for 30 min with 10% normal goat serum (S-1000, RRID:AB_2336615, Vector laboratories) and incubated overnight in rabbit anti-PREP primary antibody (1:500; custom made monoclonal antibody, Thermo Fisher Scientific). Followed by goat anti-rabbit secondary antibody for 2 h (1:500; #31460, RRID: AB_228341, Thermo Fisher Scientific).

### Proteinase K treatment

PK protocol to remove soluble proteins in sections was performed as described in^18^. Shortly, sections were digested with 10 μg/ml PK (#V3021, Promega, Madison, USA) in TBS-T for 10 min at 55 °C. The sections were post-fixed with 4% PFA for 10 min and processed for aSyn oligomer specific immunostaining with the same primary and secondary antibody concentrations as described above.

### Microscopy and stereology

OD of TH, aSyn from ipsilateral and contralateral striatum and SN were determined. Digital images were scanned at 40× magnification with Pannoramic Flash II Scanner (Version 1.15.3, RRID:SCR_014424, 3DHISTECH) and three coronal sections from each mouse were processed for further analyses with Pannoramic Viewer (Version 1.15.3, 3DHISTECH). Images were converted to grayscale and inverted, line analyses tools for striatum or freehand for SN in ImageJ (1.48b; RRID:SCR_003070, NIH, USA) were used to measure the OD of immunoreactivity.

p129-aSyn immunohistochemical sections were imaged and average particle area and numbers per section were quantified using Image-Pro Plus software (Media Cybernetics, Inc., Rockville, MD, USA), four representative SN sections per brain were used.

The number of TH+ cells in SNpc was estimated using the optical fractionator method in combination with the dissector principle and unbiased counting rules^57^. The SNpc was analyzed with a Stereo Investigator platform (MicroBright-Field, RRID:SCR_002526, Magdeburg, Germany) attached to an Olympus BX51 microscope (Olympus Optical, Tokyo, Japan) as described previously^18^. From each animal, three sections from the central portion of the SNpc were selected for quantitative analysis. Grid size was 100 × 80 µm and the counting frames were 60 × 60 µm. The coefficient of error was between 0.05 and 0.10^58^.

The number of aSyn oligomer specific particles in SN was estimated using the optical fractionator method in combination with the dissector principle and unbiased counting rules^57^. From each animal, four representative sections from SN were selected for quantitative analysis as described previously^18^. Grid size was 120 × 120 µm and the counting frames were 60 × 60 µm large. The average coefficient of error for each region was in range of 0.05 to 0.1^58^.

High magnification images were acquired with Qimaging 2000R camera (Qimaging, Canada) attached to Olympus BX51 microscope with Olympus Microscope Objective Lens UPlanApo 20×/0.5 and100x/1.35 Oil Iris and processed with Adobe Photoshop CS6 (Version 13.0 × 64, RRID:SCR_014199).

### No-net-flux microdialysis

Extracellular concentration of striatal DA was measured by no-net-flux microdialysis that was performed with PREPko mice and wt littermates 14–15 weeks after the injection of viral vectors as described in^11^. After the microdialysis experiment, the brains were removed and processed for tissue HPLC analysis.

### HPLC tissue analysis

Striatal tissue samples were punched below corpus callosum +0.74 mm from bregma to 2 mm depth by using sample corer (i.d. of 2 mm) with a plunger (Stoelting Co, Wood Dale, IL, USA) on a cryostat (Leica CM3050). Tissue processing was done as earlier described in^59^. The concentration of DA, its metabolites, DOPAC and HVA, 5-HT, its metabolite 5-HIAA and GABA in the tissue samples of striatum were analyzed with an HPLC as earlier described in^18^. Concentrations were calculated as nanograms per milligram of brain tissue.

## ***In vitro*** and cell assays

### Cell lines

HEK-293 or stable PREPko cells generated in HEK-293 background were used throughout the whole study. PREPko cells were generated using CRISPR-cas9n plasmid (pSpCas9n(BB)-2A-Puro (PX462) V2.0; Addgene plasmid; #62987) targeted at 3^rd^ exon of the *PREP* gene^60^. Two oligonucleotides for CRISPR guide A (5′atggcacagtaatctt) and B (5′cttgagcagtgtccca) were designed and annealed separately. Backbone PX462 was digested with BbsI restriction enzyme (#R0539S, New England Biolabs, Ipswich, USA) and ligated with Ligate-IT (USB® Ligate-IT™ Rapid Ligation Kit, Affymetrix, Santa Clara, USA).

HEK-293 cells were cultured in full Eagle’s medium with an additional 10% (v/v) FBS (Invitrogen, Carlsbad, USA), 1% (v/v) L-glutamine-penicillin-streptomycin solution (Lonza, Basel, Switzerland). PREPko cells were cultured in Dulbecco’s modified Eagle’s medium with an additional 20% (v/v) FBS (Invitrogen), 1% (v/v) L-glutamine-penicillin-streptomycin solution (Lonza) at 37 °C and 5% CO_2_, water-saturated air.

### Induction of aSyn aggregation

For WB, cells were seeded in a 6-well plate with the density 200,000 cells/well or 400,000 cells/well for oxidative stress conditions and allowed to attach overnight. Thereafter, the cells were transfected with aSyn, GFP, aSyn + GFP or aSyn + PREP. Lipofectamine 3000 (L3000015; Thermo Fisher Scientific) was used as a control. Non-stressed cells were grown for 72 hours. In oxidative stress groups 24 hours after plasmid transfection, the aggregation process of aSyn was induced by adding 100 mM H_2_O_2_ and 10 mM FeCl_2_ in cell culturing medium, adapted from^15^. Cell lysis and fractionation were performed as described below.

### Cell viability assay

Cells were plated with the density of 10,000 cell/well in 96-well plate, the next day transfected with aSyn, GFP and aSyn + PREP and thereafter incubated for 24 h. Lipofectamine 3000 was used as a control. The cells were exposed to oxidative stress for 48 hr as described above (See Materials and Methods). To assess cell viability standard MTT test was performed as previously described in^61^.

### Reactive oxygen species detection

ROS in cells were measured using DCFDA Cellular ROS Detection Assay Kit (ab113851, AbCam) after induction of oxidative stress as described above. After 48 hours, DCFDA treatment was performed according to the manufacturer’s instructions. Fluorescence signal was adjusted to the total protein amount.

### Cell fractionation and Western blot

WB was used to detect aSyn accumulation, autophagy and stress response markers from cellular fractions. The cells were fractioned in TBS for soluble aSyn, in Triton X-100 for membrane-bound aSyn and in SDS buffer for SDS soluble and insoluble aSyn conformations as described in^26,62^. We performed additional aSyn + GFP transfection but as this condition was too toxic for the cells it was excluded from the further analyses (Figs 6 and 7). Observed toxicity probably was due to increased aggregate prone protein overload^63^. Before WB, the protein concentrations of TBS fraction were measured by BCA method (Bio-Rad, Hercules, CA, USA), and the samples were loaded to 4–20% TGX gels (Bio-Rad) with equal protein amounts. Triton X-100 fraction was partially freeze dried (Heto Lyopro 3000, Copenhagen, Denmark) in order to load whole Triton X-100 lysate onto WB. The protein amounts from Triton X-100 and SDS-fractions were correlated to total protein amounts as assessed by Ponceau S Staining. Standard SDS-PAGE techniques were used, and the membranes were incubated +4 °C overnight in 5% skim milk in TBS-T. The following primary antibodies were used: goat anti-aSyn (phospho S129) (1:500; ab51253, RRID:AB_2270761, AbCam), sheep anti-aSyn (1:1000; ab6162, RRID:AB_2192805, AbCam), mouse anti-SQSTM1/p62 (p62, 1:5000; ab56416, RRID:AB_945626, AbCam), rabbit anti-microtubule associated protein light chain 3B I-II (LC3BI-II, 1:1000; #L7843), rabbit anti-beclin1 (1:2000; ab207612, RRID:AB_2692326 AbCam), rabbit oxidative stress defense cocktail (1:250; ab179843, RRID:AB_2716714, AbCam) mouse anti-GAPDH (1:2000; MAB374, RRID:AB_2107445, Millipore), rabbit anti-β-actin (1:2000; ab8227, RRID:AB_2305186, AbCam). After overnight incubation, the membranes were washed and incubated with HRP-conjugated secondary antibodies for 2 h in room temperature for SQSTM1/p62, goat anti-mouse HRP (dilution 1:2000 in 5% milk, #31430; Thermo Fischer Scientific); for aSyn, donkey anti-sheep HRP (dilution 1:2000 in 5% milk; ab6900, RRID:AB_955452, AbCam), aSyn p129, LC3BI-II, beclin1, β-actin, goat anti-rabbit (dilution 1:2000; Product #31463, Thermo Fisher Scientific). The images were captured using the C-Digit imaging system (Licor, Lincoln, USA). Six independent WB experiments were performed. ImageJ was used for analyzing bands, and the OD values were calculated by comparing the OD value to the corresponding β-actin OD values.

### Proteasome activity assay

For determining chymotrypsin-like 20S proteasomal activity, the protocol based on Suc-Leu-Leu-Val-Tyr-AMC (#I-1395, Bachem, Bubendorf, Switzerland) substrate was used as described in^17^. In brief, cells were lysed in buffer for 20S activity. After 1 hr incubation at 37 °C with substrate, fluorescence was read at 355/460 nm with Victor 2 well-plate reader (PerkinElmer, Waltham, USA). Proteolytic activity was expressed as the amount of free AMC/min ∗ mg protein.

### Autophagic Flux measurements

To assess the autophagic flux PREPko and HEK-293 cells were treated with 10 and 50 nM concentration of bafilomycin A1 for 4 hrs. Dimethyl sulfoxide (DMSO) served as a control. Cells were lysed in ice cold modified RIPA buffer (50 mM Tris HCl pH 7.4, 1% NP-40, 0.25% sodium deoxycholate, 150 mM NaCl) and WB for LC3B was performed as describe above (see Materials and Methods).

### aSyn ELISA from cell culture supernatant

aSyn levels in cell culture medium of PREPko and HEK-293 cells were measured after cell transfection either with aSyn or aSyn + PREP plasmids and induction of aSyn aggregation as described above (see Materials and Methods). Human aSyn ELISA Kit (ab210973; AbCam) was used according to the manufacturer’s instruction. Fluorescence was red at 450 nm with Victor 2 well-plate reader (PerkinElmer). aSyn amount in medium was adjusted to the cell protein concentration in cell lysates, pmol/mg*protein.

### Statistical Analysis

Statistical analyses were performed using either GraphPad Prism (version 6.07, GraphPad Software, Inc., San Diego, USA) or SPSS Statistics (Version 22.0.0.1 IBM Corporation, Armonk, USA) tools. Statistical tests that were used were Student’s t-test and two-way ANOVA with Bonferroni’s post hoc comparison for behavioral assessment and one-way, two-way and three-way ANOVA with Tukey’s post hoc comparison for *in vitro* and cell data. Data are presented as mean ± SEM. Statistically significant differences were considered at p < 0.05.

## Electronic supplementary material

Supplementary figures
