# Supplementary material for: Removal of prolyl oligopeptidase reduces alpha-synuclein toxicity in cells and in vivo
Source: Sci Rep. 2018 Jan 24;8:1552. doi: 10.1038/s41598-018-19823-y (PMC5784134; doi:10.1038/s41598-018-19823-y)
Supplement: Supplementary file 1 — Supplementary figures [file 41598_2018_19823_MOESM1_ESM.pdf]

SUPPLEMENTARY MATERIAL

Removal of prolyl oligopeptidase reduces alpha-synuclein toxicity in cells and in vivo

Reinis Svarcbahs[1], Ulrika H Julku[1], Susanna Norrbacka[1], Timo T Myöhänen[*1]

[1]University of Helsinki, Division of Pharmacology and Pharmacotherapy, Viikinkaari 5E, P.O. Box 56, 00014 University of Helsinki, Finland

[*]Corresponding author: Timo T Myöhänen

University of Helsinki, Division of Pharmacology and Pharmacotherapy, Viikinkaari 5E, P.O. Box 56, 00014 University of Helsinki, Finland. timo.myohanen@helsinki.fi, Tel: +358-50-4480769

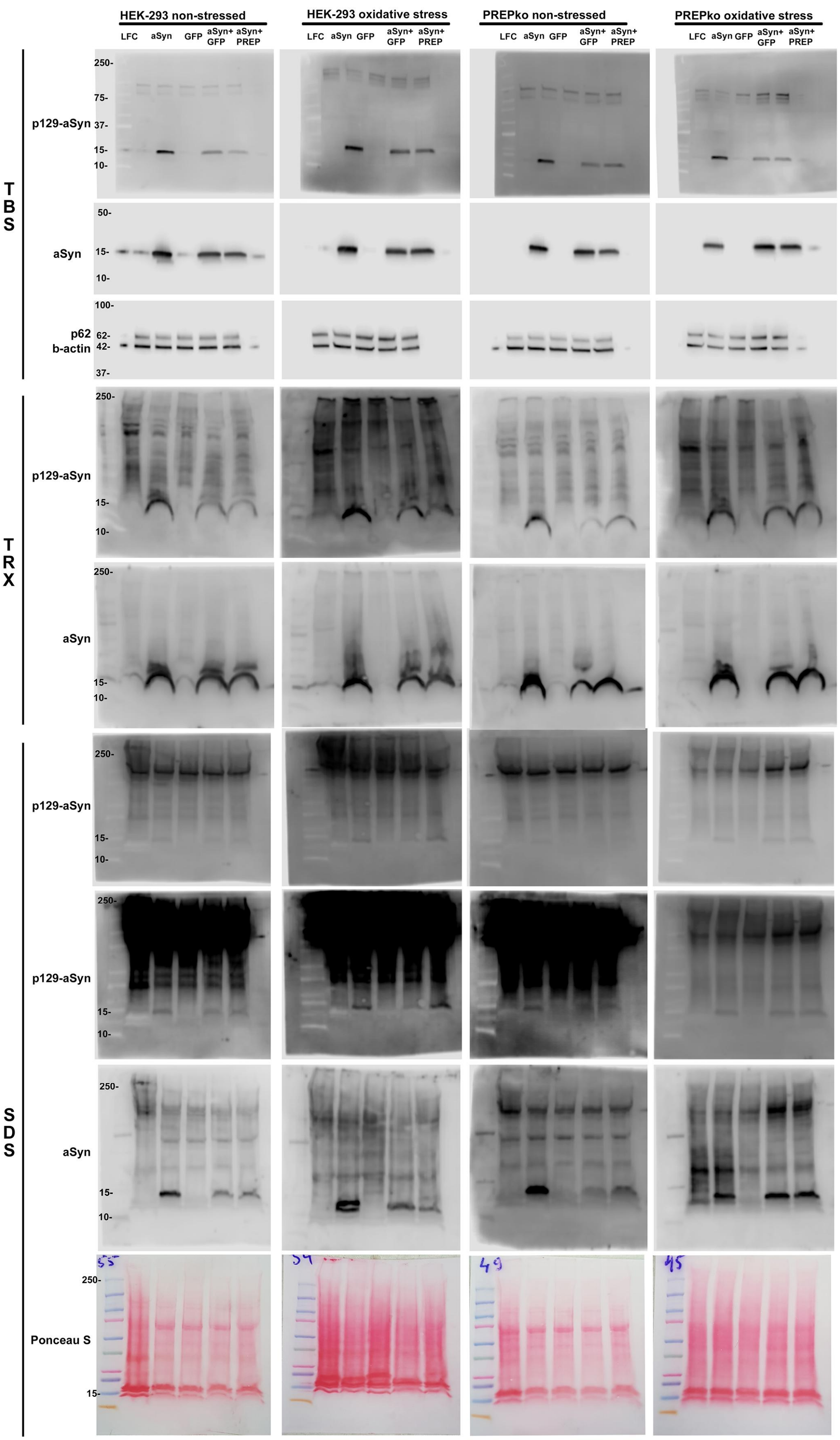
HEK-293 non-stressed
HEK-293 oxidative stress
PREPko non-stressed
PREPko oxidative stress
LFC
aSyn
GFP
aSyn+ GFP
aSyn+ PREP
TBS
p129-aSyn
aSyn
p62
b-actin
TRX
p129-aSyn
aSyn
SDS
p129-aSyn
p129-aSyn
aSyn
Ponceau S
250-
75-
37-
15-
10-
50-
100-
62-
42-

Supplement Figure S1. WB images of aSyn distribution between soluble (TBS), membrane bound (TRX) and insoluble fractions (SDS). HEK-293 and PREPko cells were transfected with Lipofectamine 3000 control, aSyn, GFP, aSyn+GFP or aSyn+PREP and treated with oxidative stress for 48 hrs. Distribution of aSyn and p129-aSyn between soluble, membrane bound and insoluble fraction and autophagy markers in soluble fraction were measured with Western blot. Insoluble p129-aSyn fractions are presented at two exposures.

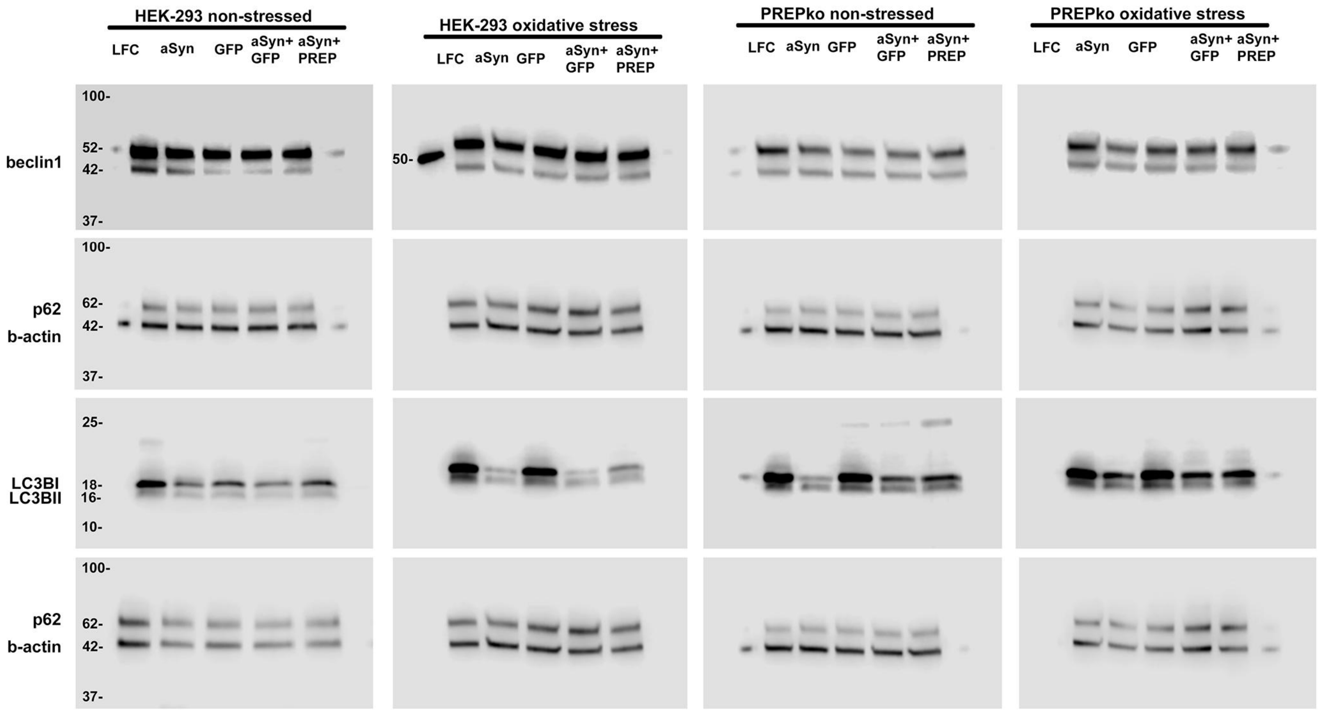

Supplement Figure S2. WB images of autophagy markers in soluble fractions of HEK-293 and PREPko cells. Cells were transfected with Lipofectamine 3000 control, aSyn, GFP, aSyn+GFP or aSyn+PREP and treated with oxidative stress for 48 hrs.

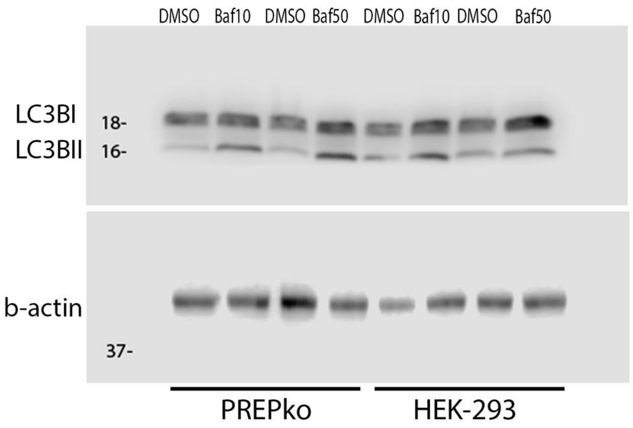

Supplement Figure S3. WB images of LC3B markers in HEK-293 and PREPko cells after Bafilomycin A1 treatment. HEK-293 and PREPko cells were treated with 10 or 50 nM Bafilomycin A1 (Baf) or DMSO control. Changes in autophagic flux (LC3BII levels) were measured with Western blot

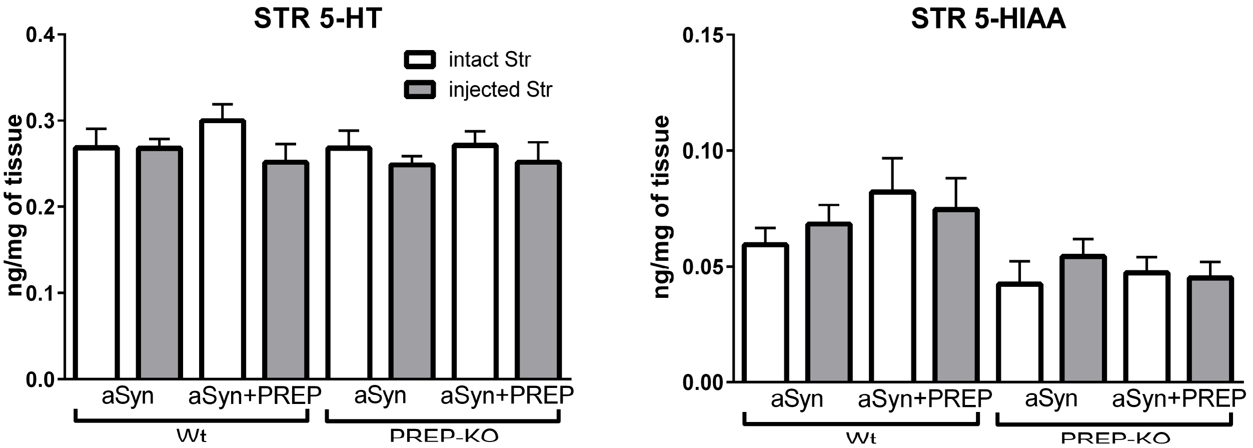

Supplement Figure S4. 5-HT and 5-HIAA levels in wt and PREPko mice were not altered. Tissue concentrations of neurotransmitter and its metabolite were measured 14-15 weeks post-injection by tissue HPLC analysis. Striatal 5-HT (A) and its metabolite 5-HIAA (B) were not changed. Bars represent mean ± SEM.
